# Transdifferentiated Human Vascular Smooth Muscle Cells are a New Potential Cell Source for Endothelial Regeneration

**DOI:** 10.1038/s41598-017-05665-7

**Published:** 2017-07-17

**Authors:** Xuechong Hong, Andriana Margariti, Alexandra Le Bras, Laureen Jacquet, Wei Kong, Yanhua Hu, Qingbo Xu

**Affiliations:** 1 Cardiovascular Division, BHF Centre for Vascular Regeneration, King’s College London, London, UK; 2 Centre for Experimental Medicine, School of Medicine, Dentistry and Biomedical Sciences, Queen’s University Belfast, Belfast, UK; 3 School of Basic Medical Sciences, Peking University; Key Laboratory of Molecular Cardiovascular Science, Ministry of Education, Beijing, China

## Abstract

Endothelial dysfunction is widely implicated in cardiovascular pathological changes and development of vascular disease. In view of the fact that the spontaneous endothelial cell (EC) regeneration is a slow and insufficient process, it is of great interest to explore alternative cell sources capable of generating functional ECs. Vascular smooth muscle cell (SMC) composes the majority of the vascular wall and retains phenotypic plasticity in response to various stimuli. The aim of this study is to test the feasibility of the conversion of SMC into functional EC through the use of reprogramming factors. Human SMCs are first dedifferentiated for 4 days to achieve a vascular progenitor state expressing CD34, by introducing transcription factors OCT4, SOX2, KLF4 and c-MYC. These SMC-derived progenitors are then differentiated along the endothelial lineage. The SMC-converted ECs exhibit typical endothelial markers expression and endothelial functions *in vitro*, *in vivo* and in disease model. Further comprehensive analysis indicates that mesenchymal-to-epithelial transition is requisite to initiate SMCs reprogramming into vascular progenitors and that members of the Notch signalling pathway regulate further differentiation of the progenitors into endothelial lineage. Together, we provide the first evidence of the feasibility of the conversion of human SMCs towards endothelial lineage through an intermediate vascular progenitor state induced by reprogramming.

## Introduction

Vascular endothelial cells (ECs) align the most inner layer of vascular structure and serve not only as the frontline barrier between blood and tissue, but also as a key regulator of vascular homeostasis. Endothelial dysfunction triggers a cascade of pathological changes that leads to the development of atherosclerosis and subsequent macro- and micro-vascular diseases^1^. Since spontaneous EC regeneration is a slow and insufficient process, it is of great interest to explore alternative cell sources that are capable of generating functional ECs. Stem cell-based endothelial regeneration strategies have been explored for the purpose of therapeutic angiogenesis to restore blood perfusion to ischemic tissue, or for the construction of tissue-engineered vascular graft. However, the optimal cell source to generate functional endothelial-like cells is still under discussion^2, 3^. Recent advances in cell lineage conversion techniques remarkably extend the cell candidates for vascular regeneration purpose.

Human vascular smooth muscle cell (SMC) is an important vascular cell type that underlies the endothelium and composes the majority of the vessel wall. In response to endothelial injury, SMCs proliferate and migrate towards tunica intima and accumulate underneath the injured endothelium^4^. SMCs retain a certain degree of phenotypic plasticity in response to various stimuli. SMCs can exhibit phenotypes of macrophage or mesenchymal stem cell during atherosclerosis progression^5, 6^. Developmentally, SMC and EC are both of mesodermal origin. SMCs can originate from multiple types of progenitor cells during embryonic and postnatal development, among which vascular progenitors expressing CD34 or Flk1 that can give rise to both SMCs and ECs^7–10^. Along the differentiation of induced pluripotent stem (iPS) cells towards cardiovascular cells, a mesoderm progenitor cell state is firstly reached, which can further be differentiated into endothelial- or smooth muscle-like cells^11^. Evidence of common progenitors for EC and SMC implies that vascular SMCs can be ontogenetically more related to EC compared to other cell types such as fibroblasts that have been used in many transdifferentiation studies to induce endothelial-like cells^12–15^. Taken together, it is of particular interest to investigate the feasibility of SMC serving as a potential cell source to generate endothelial-like cells.

Currently, there are two reprogramming strategies based on the use of transcription factors to achieve cell-lineage conversion. One strategy consist in introducing various combinations of transcription factors specific of the target cell type to directly drive the cell lineage switch. The ectopic expression of different sets of transcription factors has already successfully reprogrammed fibroblasts into many different somatic cell types including ECs^14, 16–18^. However, cells converted with this method sometimes tend to keep the epigenetic memory of the original cell type which affects the newly acquired cell identity^19^. Another approach is based on the use of induced Pluripotent Stem (iPS) cell generating transcription factors such as *Oct4*, *Sox2*, *Klf4*, or *Nanog* to erase the starting cell’s lineage-specific signatures^20, 21^. Cells therefore revert to an intermediate plastic state which permits further manipulation and new lineage commitment towards the desired cell types^22, 23^. Several studies have used this strategy to convert fibroblasts towards an endothelial fate^12, 13^. A recent study reduced the number of reprogramming factors to only *Oct4* and *Klf4* to efficiently generate functional endothelial-like cells from human fibroblasts^15^. Considering that SMC and EC could be derived from common vascular progenitors, it appears relevant to use a transdifferentiation strategy consisting in firstly de-differentiating the SMCs back to an intermediate progenitor state with iPS-generating transcription factors and then re-differentiating them towards the endothelial lineage.

In this study, we provide the first evidence of the successful conversion of human SMC towards the endothelial lineage based on a combined protocol of short time dedifferentiation with four iPS-generating transcription factors (*OCT4*, *SOX2*, *KLF4*, and *c-MYC*) and differentiation under endothelial inductive culture condition. The SMC-derived ECs not only acquire a panel of endothelial gene expression characteristics, but also display endothelial functions *in vitro* and *in vivo*. More importantly, the SMC-derived ECs exhibit therapeutic angiogenic potential in a mouse hindlimb ischemia model and the capacity to reendothelialize the decellularized mouse aortic vascular graft through an *ex vivo* circulation bioreactor system. In addition, we explore possible mechanisms underlying SMC to EC conversion and reveal the involvement of mesenchymal-to-epithelial transition and members of Notch signalling pathway. Our study provides a novel potential strategy for the purpose of endothelial regeneration.

## Results

### Conversion of Human Vascular Smooth Muscle Cells (SMCs) towards endothelial lineage through short term dedifferentiation using reprogramming factors

First, we verified that the human umbilical artery SMCs (UASMCs) used in this study comply with commonly recognized SMC features^24^. Compared to human fibroblasts and Human Umbilical Vein Endothelial Cells (HUVECs), UASMCs express a high level of typical SMC markers including α-SMA, SM22α, Calponin, have no expression of EC marker CD144 (also known as VE-Cadherin) and have minimal expression of FSP-1(Supplementary Fig. 1a). The expression of SMC markers is stable, albeit cell passaging *in vitro* (Supplementary Fig. 1b). Immunofluorescence staining of UASMCs shows the representative staining patterns for cytoskeletal and contractile proteins: α-SMA, SM22α, and Calponin (Supplementary Fig. 1c).

**Figure 1:**
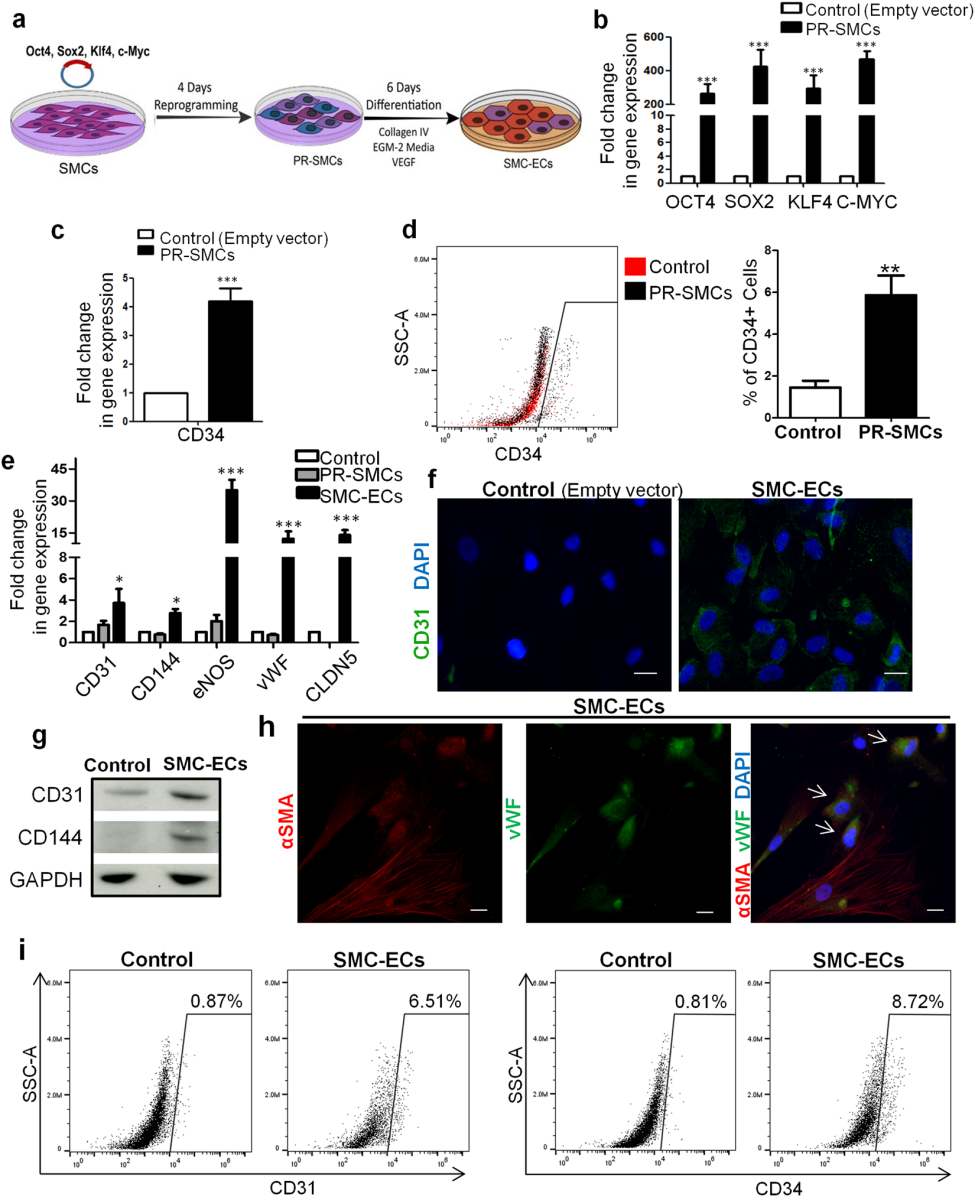
Conversion of human SMCs toward endothelial-like cells through short term dedifferentiation using reprogramming factors. (**a**) Schematic protocol of the conversion of SMCs towards endothelial-like cells. SMCs were transfected with lentivirus vector containing the four reprogramming factors Oct4, Sox2, Klf4, c-Myc and then maintained in the reprogramming media for 4 days. Partially reprogrammed SMCs (PR-SMCs) were then differentiated towards endothelial lineage by culturing with endothelial-inductive condition. (**b**) Real-time PCR showed the induction of the four reprogramming factors at the mRNA level of PR-SMCs. Control group refers to the SMCs transfected with empty lentivirus vector and maintained under the same reprogramming conditions. (***p < 0.001 by Student’s *t* test, n = 4) (**c**) Real-time PCR showed an upregulation of progenitor marker CD34 in PR-SMCs. (***p < 0.001 by Student’s *t* test, n = 4) (**d**) Flow cytometry analysis of CD34 positive cells in PR-SMC population. (*p < 0.05 by Student’s *t* test, n = 3) (**e**) Real-time PCR results revealed an overall induction of the endothelial markers CD31, CD144, eNOS, vWF and Claudin5 (CLDN5) at the mRNA level in SMC-derived endothelial like cells (SMC-ECs). (*p < 0.05, ***p < 0.001 by Student’s *t* test, n = 5). Control group refers to the SMC transfected with empty lentiviral vector that underwent the same reprogramming and differentiation protocol. (**f**) Representative images of the immunofluorescence staining for CD31 of SMC-ECs. (Scale Bar: 25 μm) (**g**) Western blot analysis showed the expression of CD31 and CD144 at the protein level of SMC-ECs. (**h**) By immunofluorescence staining, white arrows indicated the cells that started to express vWF were the cells that lost the typical filamentous αSMA expression in the SMC-ECs population. (Scale bar: 25 μm) (**i**) Flow cytometry analysis exhibited increased CD31, CD34 expression in SMC-ECs compared to control group. Western blots were cropped for clarity. Examples of uncropped blots are found in Supplementary Figure 15.

To achieve the conversion of SMCs to ECs, we adopted a short term reprogramming strategy to induce SMCs back to a dedifferentiated state as the first step, by ectopic overexpression of the four transcription factors *OCT4*, *SOX2*, *KLF4*, *c-MYC* (Fig. 1a). SMCs were transfected with a lentivirus encoding OCT4, SOX2, KLF4, and c-MYC or empty vector as control and then maintained in the reprogramming condition. Transduced SMCs overexpressed the corresponding mRNAs to adequate levels (Fig. 1b). After four days, partially reprogrammed (dedifferentiated) SMCs (PR-SMCs) displayed distinct morphological changes compared to the empty vector control group (Supplementary Fig. 2a). We named the SMCs after four day reprogramming as PR-SMCs because a four day dedifferentiation time is much shorter than general time, 21–28 days, to induce fully reprogrammed iPS cells from human SMCs or human fibroblasts^25, 26^. PR-SMCs lost the expression of typical SMC markers (Supplementary Fig. 3a). In contrast, we observed an upregulation of vascular progenitor marker CD34 in the PR-SMCs population at the mRNA level (Fig. 1c). Previous studies showed that CD34 can be considered as an important marker for vascular progenitor cells that can give rise to both endothelial- and smooth muscle-like cells^9, 13, 27, 28^. Flow cytometry analysis demonstrated that there are 5.86% ± 0.9% CD34 positive cells among the PR-SMCs population (Fig. 1d). At this point, CD34 positive PR-SMCs didn’t co-express neither endothelial marker CD31 nor progenitor markers KDR, C-KIT or PDGFRα (Supplementary Figure 4a–e). No significant increase of CD34 or CD31 induction can be observed when the reprogramming time was prolonged to 6 days (Supplementary Figure 4f,g). PR-SMCs did not express pluripotency markers or hematopoietic markers after 4 day dedifferentiation at the mRNA or protein level (Supplementary Fig. 5a,b). Thus, we concluded that a vascular progenitor state has been induced from SMCs after four day dedifferentiation.

To drive the differentiation of PR-SMCs towards the endothelial lineage, cells were treated with endothelial-inductive culture condition based on established protocols used for the differentiation of pluripotent cells or progenitor cells to ECs^12, 29^. PR-SMCs were seeded on Collagen IV coated petri-dish and maintained in EGM-2 media supplemented with 25 ng/ml of VEGF. With these conditions, SMCs converted Endothelial-like Cells (SMC-ECs) were generated and further analysis was performed following six days of differentiation (Fig. 1a). SMCs transfected with empty lentivirus vectors that underwent the identical reprogramming and differentiation process were used as the control group. SMC-ECs demonstrated altered morphology, appearing shorter, rounder and flatter than control cells (Supplementary Fig. 2b). At this point, a panel of typical endothelial markers including CD31 (also known as PECAM-1), CD144, eNOS, vWF and Claudin5 (CLDN5) were significantly upregulated in SMC-ECs as shown by real-time PCR (Fig. 1e). Immunofluorescence staining showed that SMC-ECs were positive for CD31 and vWF expression (Fig. 1f, Supplementary Fig. 6). Western blot analysis confirmed the expression of specific endothelial markers CD31 and CD144 at the protein level (Fig. 1g). Moreover, immunofluorescence staining of SMC-ECs population also showed that the cells that expressed vWF were the cells that lost the α-SMA typical cytoskeletal staining pattern (Fig. 1h). Flow cytometric analysis confirmed the increase of CD31 (6.51%) and CD34 (8.72%) positive cells in SMC-EC population compared to control (<1%) (Fig. 1i). Altogether, these results indicated that PR-SMCs have the potential to commit to the endothelial lineage in response to endothelial-inductive stimuli.

To verify that the four reprogramming factors were the main force to initiate SMC to endothelial lineage conversion, we employed an alternative method to ectopically overexpress *OCT4*, *SOX2*, *KLF4* and *c-MYC* in SMCs. SMCs were transfected with a linearized pCAG2LMKOSimO plasmid encoding the four factors or with an empty plasmid as control and then treated using an identical protocol of reprogramming and differentiation to the one described above (Supplementary Fig. 7a). Analysis revealed the upregulation of CD34 after four days reprogramming (Supplementary Fig. 7b) and the induction of typical endothelial markers after six days of differentiation (Supplementary Fig. 7c,d), which were the same as using the lentivirus to overexpress the four factors. We also confirmed the reproducibility of the protocol with a second batch of UASMC. Upregulated CD34 expression after 4 days dedifferentiation and increased endothelial markers expression after endothelial differentiation could be observed (Supplementary Fig. 8a,b).

### CD34 positive cells selected from PR-SMCs give rise to a more enriched endothelial-like cell population

**Figure 2:**
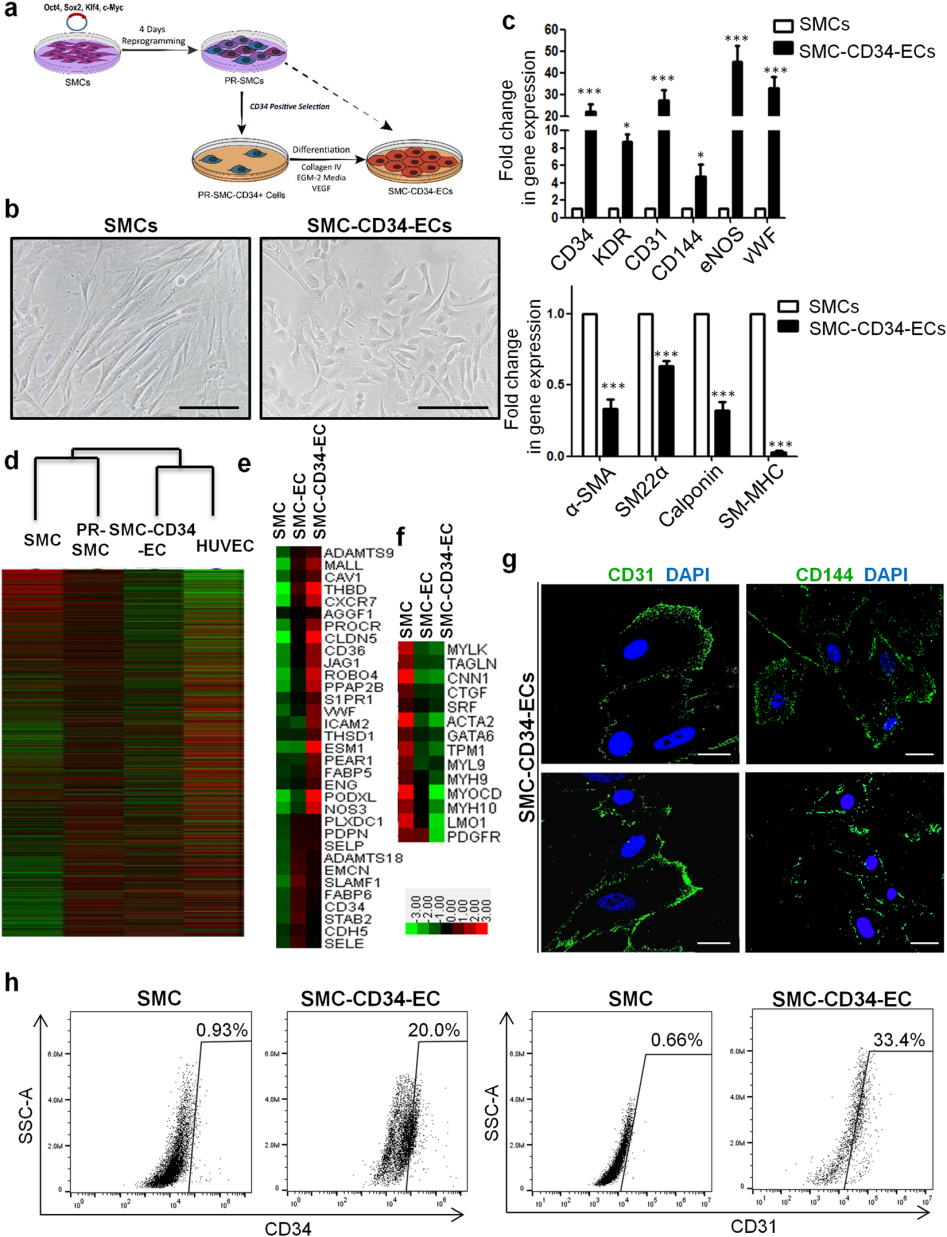
CD34 positive cells selected from PR-SMCs can differentiate into a more enriched endothelial-like cell population. (**a**) Schematic protocol of selecting CD34 positive cells from PR-SMCs and further differentiation towards the endothelial lineage. (**b**) CD34 positive cells-derived endothelial cells (SMC-CD34-ECs) displayed an endothelial-like monolayer compared to SMCs. (Scale Bar: 100 μm) (**c**) Real-time PCR result showed a strong induction of endothelial markers including KDR, CD31, CD144, eNOS, vWF and a strong suppression of SMC markers α-SMA, SM22α, Calponin and SM-MHC. (*p < 0.05, ***p < 0.001 by Student’s *t* test, n = 4) (**d**) Hierarchical clustering analysis of global gene expression from RNA-Seq of SMCs, PR-SMCs, SMC-CD34-ECs and HUVECs showed a shift of the overall gene expression towards ECs. (**e**,**f**) Heat map of EC and SMC enriched gene expression changes among SMC, SMC-ECs and SMC-CD34-ECs based on the RNA-Seq results. Colour bar indicates gene expression in scale. (**g**) Immunofluorescence staining of SMC-CD34-ECs revealed the typical junctional staining pattern of the endothelial markers CD31 and CD144. (Scale bar: 25 μm) (**h**) Flow cytometry analysis evaluated the increase of the progenitor marker CD34 and endothelial marker CD31 in SMC-CD34-ECs compared to SMCs.

Given the emergence of CD34 positive cells among PR-SMCs, along with the fact that CD34 can be considered as a marker of vascular progenitor cells, we wanted to test whether CD34 positive cells selected from the heterogeneous PR-SMCs population can give rise to a more enriched endothelial population. CD34 selection has been applied in many endothelial differentiation protocols in order to generate a mesodermal vascular progenitor or endothelial progenitor population for subsequent differentiation towards ECs^13^. CD34 positive cells were sorted from PR-SMCs using anti-CD34 antibody-coupled magnetic microbeads, then seeded on Collagen IV coated petri-dish and cultured in the same endothelial-inductive media as used before (25 ng/ml VEGF-supplemented EGM-2) (Fig. 2a). After ten days of differentiation, CD34 positive cells formed an endothelial-like monolayer (Fig. 2b). CD34 positive cells selected from cells transduced with a control vector were of too little amount and could not survive. Therefore, the original SMCs population was used as control group in this session. Since a relatively small number of cells were obtained after CD34 selection, we chose day 10 as the time point for further analysis to get sufficient cell numbers. Prolonging the endothelial differentiation time of SMC-EC to 10 days did not effectively further increase endothelial marker expression (Supplementary Fig. 9).

Real-time PCR analysis showed a significant induction of a full panel of endothelial markers including KDR, CD31, CD144, eNOS, vWF and the suppression of SMC markers including α-SMA, SM22α, Calponin, SM-MHC at the mRNA level in SMC-derived CD34 positive cells converted ECs (SMC-CD34-ECs) compared to SMCs (Fig. 2c). Transcriptome sequencing (RNA-Seq) was performed on the cell populations at the different stages of the SMC to EC conversion including SMC, PR-SMC, SMC-CD34-EC and on HUVEC. These genome-wide analyses showed the global gene expression of SMC-CD34-ECs displayed an overall shift towards the human endothelial cell line (Fig. 2d). The upregulation of a cluster of endothelial enriched genes (Fig. 2e) and downregulation of a cluster of SMC genes were detected at the transcriptome level in SMC-ECs and SMC-CD34-ECs (Fig. 2f). Compared to SMC-ECs, SMC-CD34-ECs were more enriched with ECs because of the CD34 selection of PR-SMCs. Gene ontology analysis of gene upregulated in SMC-CD34-ECs compared to SMC indicated the enrichment for immune response and cell adhesion related biological processes, which are usually identified in ECs (Supplementary Fig. 10). At the protein level, following immunofluorescence staining, CD31 and CD144 displayed a typical junctional expression pattern in SMC-CD34-ECs (Fig. 2g). Furthermore, flow cytometry analysis revealed the high percentage of SMC-CD34-EC expressing CD31 (33.4%) (Fig. 2h). Based on the above findings, CD34 positive PR-SMCs represented a more optimized cell population for EC differentiation compared to the heterogeneous PR-SMC population.

### SMC-converted ECs exhibit endothelial functions *in vitro* and *in vivo*

**Figure 3:**
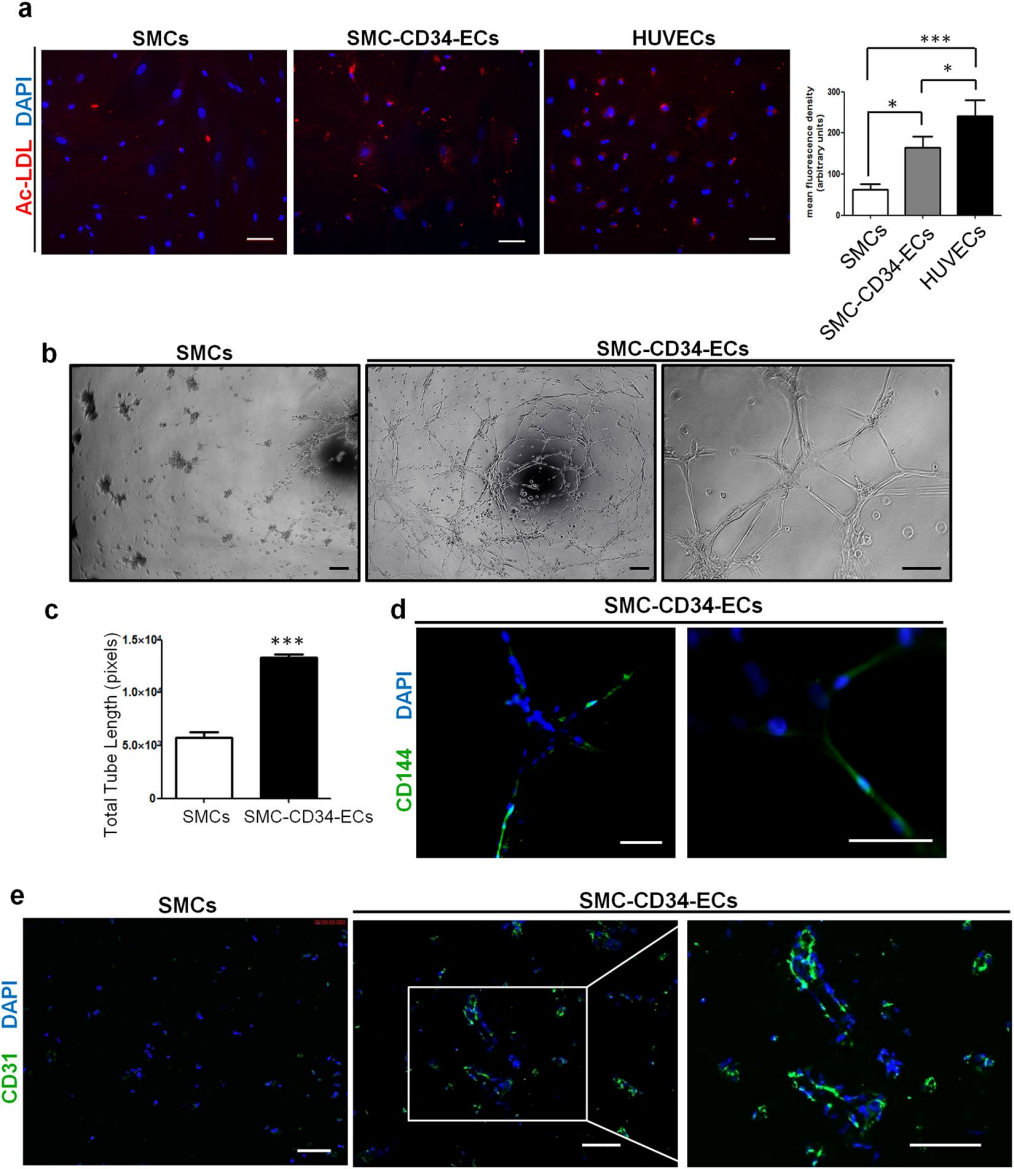
Endothelial function tests of SMC-CD34-ECs. (**a**) SMC-CD34-ECs were able to take up fluorescently labelled ac-LDL in a comparable pattern to HUVECs. (Scale bar: 100 μm) Statistical analysis of the mean fluorescence density revealed significantly higher ac-LDL uptake rate of SMC-CD34-ECs than SMCs. (**b**) *In vitro* tube formation assay was performed with SMCs and SMC-CD34-ECs. SMC-CD34-ECs showed a higher efficiency for tube structures forming compared to control SMCs. (Scale bar: 100 μm) (**c**) Quantification of total tube length showed a significant increase of SMC-CD34-ECs group compared to SMCs. (***p < 0.001 by Student’s *t* test, n = 5) (**d**) The tube forming cells were immunofluorescence stained positively for endothelial marker CD144. (Scale bar: 100 μm) (**e**) SMC-CD34-ECs formed capillary-like structures in the *in vivo* Matrigel plug assays. (Scale bar: 100 μm).

Following the phenotypic characterizations, we next evaluated the endothelial related functions of the SMC-derived ECs. SMC-CD34-ECs could effectively take up fluorescently labelled acetylated low-density lipoprotein (ac-LDL) compared to SMCs, which demonstrated their lipid metabolism regulation ability resembling mature ECs (Fig. 3a). In response to tumour necrosis factor-alpha (TNFα) stimulation, SMC-ECs started to express pro-inflammatory adhesion molecule ICAM-1 in a similar pattern with HUVECs (Supplementary Fig. 11).

To assess the vasculogenic and angiogenic potential of the transdifferentiated cells, *in vitro* tube formation assay was performed first. SMC-CD34-ECs clearly aggregated into vessel-like tube structures on Matrigel (Fig. 3b). Quantification of total tube length revealed that SMC-converted ECs exhibited a much stronger tube formation capacity compared to the SMCs (Fig. 3c). Moreover, the tube forming cells were stained positively for endothelial marker CD144 (Fig. 3d). Subsequently, a subcutaneous Matrigel plug assay in mice was performed to assess the angiogenic ability of SMC-converted ECs *in vivo*. SMC-CD34-ECs could form capillary-like structures in Matrigel plugs *in vivo* after 2 weeks (Fig. 3e). Labelling SMC-CD34-ECs with Vybrant before cell injection confirmed the contribution of injected cells to the formation of vascular-like structures in the Matrigel plugs (Supplementary Fig. 12).

### SMC-derived ECs display therapeutic angiogenic capacity in a murine hindlimb ischemia model and the ability to construct tissue-engineered vascular graft

**Figure 4:**
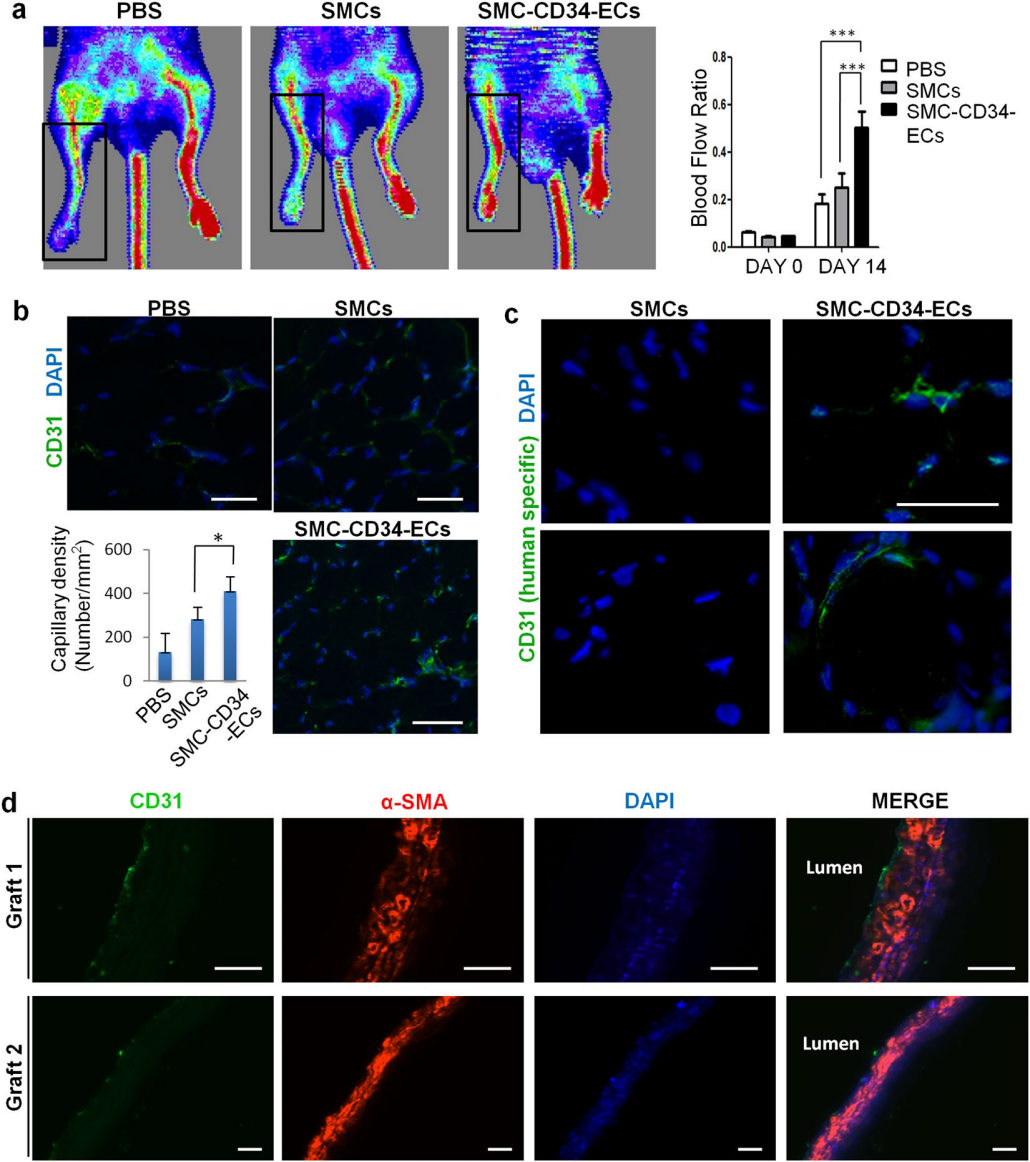
Therapeutic angiogenesis capacity of SMC-derived ECs in the mouse hindlimb ischemic model and the construction of tissue-engineering vascular grafts with SMC-CD34-ECs. (**a**) SMC-CD34-ECs, SMCs or PBS were intramuscular injected into mice ischemic hindlimb. Representative images from laser Doppler imaging exhibited that SMC-CD34-ECs injection group had a better blood flow recovery after 2 weeks. The colour scale from blue to red indicates the increase in blood flow. Quantification analysis of blood flow ratio showed significantly higher foot blood flow recovery with SMC-CD34-ECs treated group (***p < 0.001, n = 6). (**b**) Sections of ischemic area adductors muscles were immunofluorescence stained with CD31 antibody. SMC-CD34-ECs injection group showed significant clearer capillary structures compared with control cell injection group. Capillary density was quantified as the capillary number per mm^2^ (*p < 0.01, n = 6). (**c**) The incorporation of human origin SMC-CD34-ECs with *in situ* neovascularization was confirmed by human specific CD31 immunostaining. (**d**) Decellularized mouse aorta graft double seeded with SMC-CD34-ECs and SMCs showed vessel-like structures after maintained in *ex vivo* bioreactor setting for 5 days (Scale bar: 50 μm).

Besides testing endothelial function under normal physiological conditions, it is of greater importance to investigate the therapeutic value of the converted cells in a vascular disease model. The mouse hindlimb ischemia model, a commonly used animal model to evaluate pro-angiogenic therapies for ischemic diseases, was used in this study to test the therapeutic angiogenic potential of the SMC-derived ECs. The femoral artery of the mouse was ligated to create ischemia of the lower extremity. SMC-CD34-ECs, SMCs or PBS were injected intramuscularly into the ischemic hindlimbs of the mice right after the ischemic area had been created. The blood flow was evaluated immediately after the surgeries and after two weeks by laser Doppler imaging. The injection of SMC-CD34-ECs significantly improved the blood flow of ischemic hindlimb in two weeks compared to the SMCs or PBS injected groups (Fig. 4a). Ischemic area adductors muscular tissue harvested from SMC-CD34-ECs injection group showed increased capillary structures which stained positive for CD31. Quantification analysis confirmed the overall increase in capillaries in the SMC-CD34-ECs treated group (Fig. 4b). Furthermore, positive staining of human specific CD31 antibody within the capillaries proved the successful engraftment of injected human cells into *in situ* neovascularization (Fig. 4c). The angiogenesis capacity of SMC converted ECs observed in the mouse ischemic model represented a promising therapeutic alternative for treating ischemia diseases like peripheral artery disease (PAD).

Another important application of the converted ECs would be their potential to assemble into endothelium-like layer in tissue-engineered vascular graft. To test this capacity, an *ex vivo* circulation bioreactor system from our lab was used to seed the cells to the decellularized mouse aortic graft in order to generate a native vessel comparable to a vascular graft (Supplementary Fig. 13)^30–32^. Mouse aorta were first treated with chemical and mechanical methods to remove all the cells within the vascular wall. SMC-CD34-ECs were then seeded inside the decellularized vascular graft to form the endothelial layer and SMCs were seeded on the outside. Following culture in the *ex vivo* bioreactor circulation system for 5 days, the graft was harvested, sectioned and immunofluorescence stained for endothelial marker CD31 and SMC marker α-SMA. The reconstructed vascular graft displayed a vascular-like structure with the most inner endothelium-like layer formed by SMC-CD34-ECs surrounded by multiple SMC layers (Fig. 4d). SMC-converted ECs showed a strong potential to construct tissue engineered vascular graft. It is of particular interest in our case that the vascular graft can be assembled with cells of the same origin: SMCs and SMC-derived ECs, which represents a valuable prospect for regenerative medicine.

### Mesenchymal-to-epithelial transition (MET) participates in the short term reprogramming to induce vascular progenitor state from SMCs

**Figure 5:**
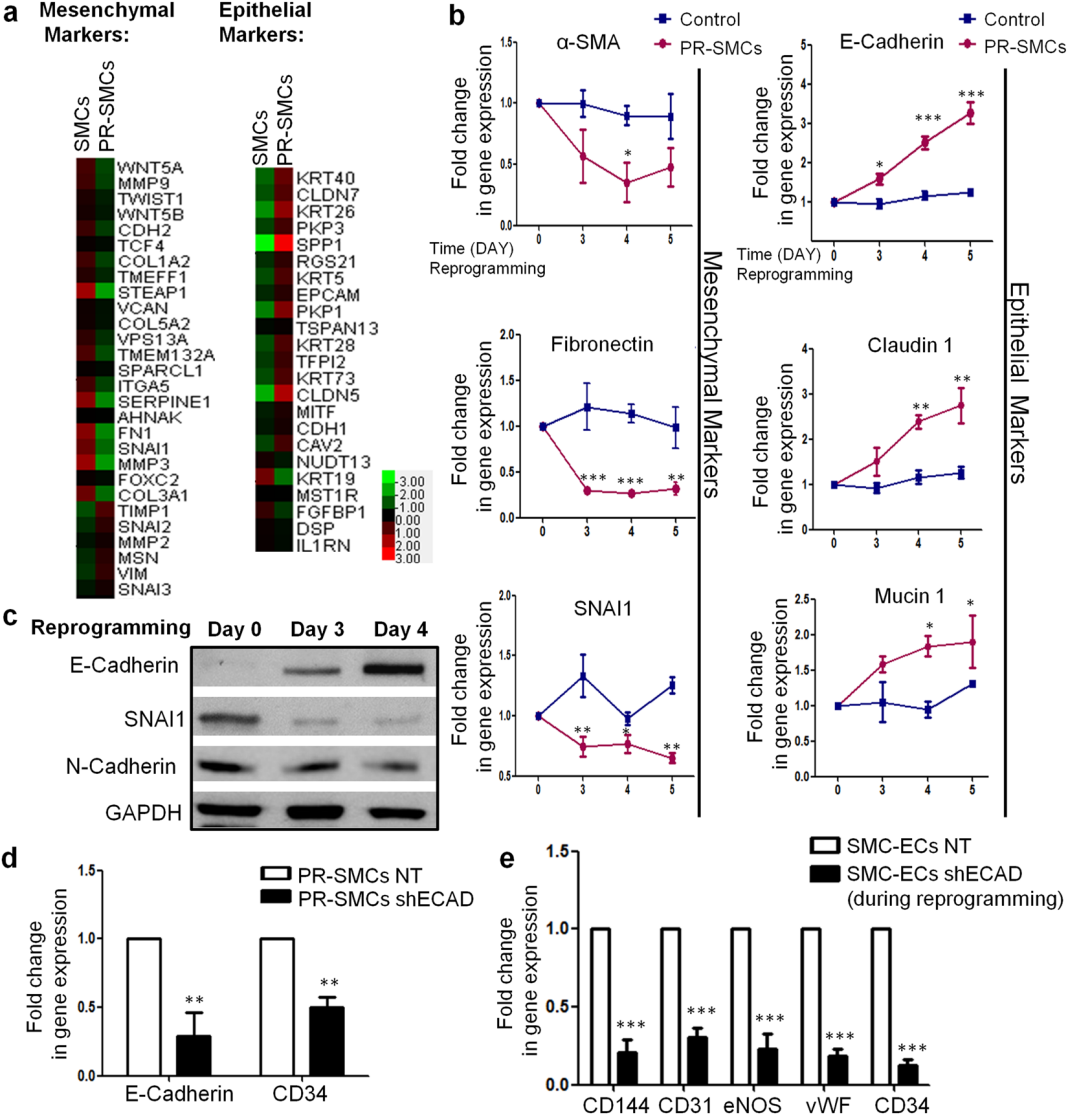
Mesenchymal-to-epithelial Transition (MET) participated in the dedifferentiation from SMCs to PR-SMCs. (**a**) Data collected from RNA-Seq showed the overall suppression of mesenchymal genes and activation of epithelial genes in PR-SMCs compared with SMCs. Color bar indicates gene expression in scale. (**b**) Real-time PCR results showed that during SMCs to PR-SMCs dedifferentiation, mesenchymal markers α-SMA, Fibronectin, SNAI1 were downregulated and epithelial markers E-Cadherin, Claudin-1, Mucin 1 were upregulated (*p < 0.05, **p < 0.01, ***p < 0.001, n = 3). Control group refers to the SMCs transfected with empty lentivirus vector and maintained under the same reprogramming conditions. (**c**) The upregulation of epithelial marker E-Cadherin and suppression of mesenchymal markers SNAI1, N-Cadherin were confirmed at protein level by western blot analysis. (**d**) Real-time PCR result indicated that knockdown E-Cadherin with shRNA at day 2 of reprogramming in concert with the downregulation of CD34 in PR-SMCs at the mRNA level (**p < 0.01, n = 3). (**e**) When subjecting PR-SMCs with or without E-Cadherin suppression to endothelial differentiation, E-Cadherin knockdown PR-SMCs showed impaired capacity of endothelial markers induction (***p < 0.001, n = 3). Western blots were cropped for clarity. Examples of uncropped blots are found in Supplementary Figure 15.

After functionally characterizing the SMC-derived EC population, we started to explore possible mechanisms underlying the conversion. It has been well established that mesenchymal-to-epithelial transition (MET) is an essential early event for successful reprogramming of somatic cells into iPS cells^33–35^. When we compared vascular progenitor state PR-SMCs to SMCs, RNA-Seq data revealed an overall MET tendency with the decrease of the typical mesenchymal markers and the increase of the epithelial markers at the transcriptome level in PR-SMCs (Fig. 5a). We then investigated the participation of MET during the vascular progenitor state induction. The expression of a panel of mesenchymal or epithelial associated markers were analysed during reprogramming from SMCs to PR-SMCs using real-time PCR analysis. Mesenchymal related genes α-SMA, Fibronectin and SNAI1 were time-dependently downregulated accompanied with the upregulation of epithelial related genes E-Cadherin, Claudin-1 and Mucin 1 (Fig. 5b). Western blots analysis confirmed the induction of E-Cadherin and the reduction of N-Cadherin and SNAI1 at the protein level (Fig. 5c). All these results suggested that MET occurred simultaneously with the induction of vascular progenitors from SMCs.

E-Cadherin is the key regulator for epithelial homeostasis. To determine whether a shift of the cell state towards epithelial-like cell is indispensable for the generation of PR-SMCs and subsequent endothelial lineage induction, we intended to knockdown E-Cadherin during reprogramming via lentiviral delivery of short hairpin E-Cadherin (shECAD). SMCs transfected with the four reprogramming factors were kept under reprogramming conditions for 2 days before transfection with shECAD or control vector. Cells were then maintained under the same reprogramming conditions for another 2 days after transfection. We observed that CD34 induction was repressed in concordance with the suppression of E-Cadherin expression in PR-SMCs (Fig. 5d), which indicated that the generation of vascular progenitor state was compromised due to the inhibition of the E-Cadherin. Accordingly, the successive endothelial differentiation capacity of PR-SMCs was jeopardized as the induction of endothelial markers was abolished at the mRNA level (Fig. 5e). Taken together, MET was identified as a key cellular process during early reprogramming for vascular progenitor generation from SMCs.

### Hairy enhancer-of-split 5 (HES5) and Jagged 1 (JAG1) are implicated in endothelial lineage derivation from PR-SMCs

**Figure 6:**
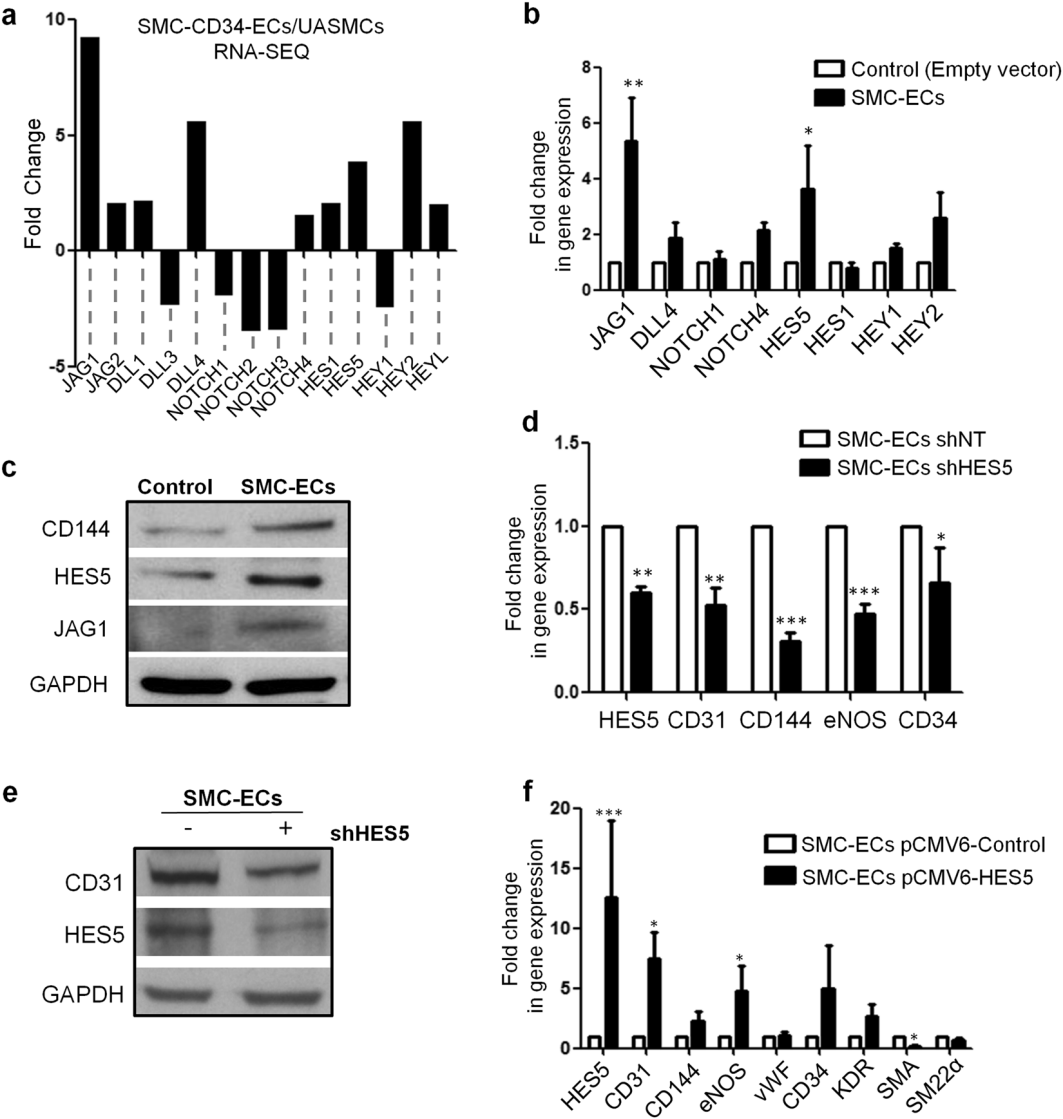
HES5 and JAG1 are implicated in endothelial lineage generation from PR-SMCs (I). (**a**) RNA-Seq data revealed the gene expression fold changes of the Notch signaling pathway members in SMC-CD34-ECs compared to SMCs. (**b**) Real-time PCR was performed to verify the upregulation of Notch members from the RNA-Seq results. JAG1 and HES5 were upregulated in converted ECs compared to the empty vector control cells (*p < 0.05, **p < 0.01 by Student’s *t* test, n = 3). (**c**) Western blot analysis showed the activation of HES5 and JAG1 along with the induction of endothelial marker. (**d**) Real-time PCR results showed the knockdown of HES5 led to the impairment of endothelial markers expression (*p < 0.05, **p < 0.01, ***p < 0.001 by Student’s *t* test, n = 3). (**e**) The suppression of CD31 with HES5 knockdown was confirmed at the protein level by western blot analysis. (**f**) Overexpressing HES5 through plasmid delivery of pCMV6-HES5 led to a further induction of the endothelial profile at the mRNA level (*p < 0.05, ***p < 0.001 by Student’s *t* test, n = 3). In this figure, all the “Control” refers to the SMCs transfected with empty lentiviral vector that underwent the same reprogramming and differentiation protocol. Western blots were cropped for clarity. Examples of uncropped blots are found in Supplementary Figure 15.

Notch signalling pathway plays an important yet complex part in regulating differentiation, proliferation, angiogenesis and other cellular processes of both EC and SMC^36^. RNA-Seq analysis revealed the upregulation of several components of the Notch pathway concurred with SMC-ECs generation (Fig. 6a). Real-time PCR was then performed to validate the RNA-Seq results (Fig. 6b). Collectively, the results particularly showed a consistent upregulation of HES5 and JAG1 during vascular progenitor to ECs differentiation. Furthermore, western blot analysis confirmed the upregulation of JAG1 and HES5 together with endothelial differentiation at the protein level (Fig. 6c). All together, we decided to look at the possible regulatory roles of HES5 and JAG1 during endothelial differentiation.

HES5 is a downstream target of the Notch pathway and functions as a transcription factor of the basic helix-loop-helix family. To elucidate the involvement of HES5 in endothelial generation from PR-SMC, we performed loss-of-function and gain-of-function analysis. Lentivirus short hairpin HES5 (shHES5) was delivered at day 3 of differentiation to suppress HES5 expression. ShHES5-mediated HES5 knockdown resulted in the reduction of endothelial markers at the mRNA and protein levels (Fig. 6d,e), which indicated that HES5 was associated with the regulation of key processes for endothelial induction. In addition, overexpressing HES5 during differentiation at day 3 via the transfection of HES5-encoding plasmid led to further augmentation of endothelial markers expression and suppression of smooth muscle marker expression (Fig. 6f). Above results indicated that HES5 played a role in facilitating the differentiation of the vascular progenitor state PR-SMC towards the endothelial lineage.

**Figure 7:**
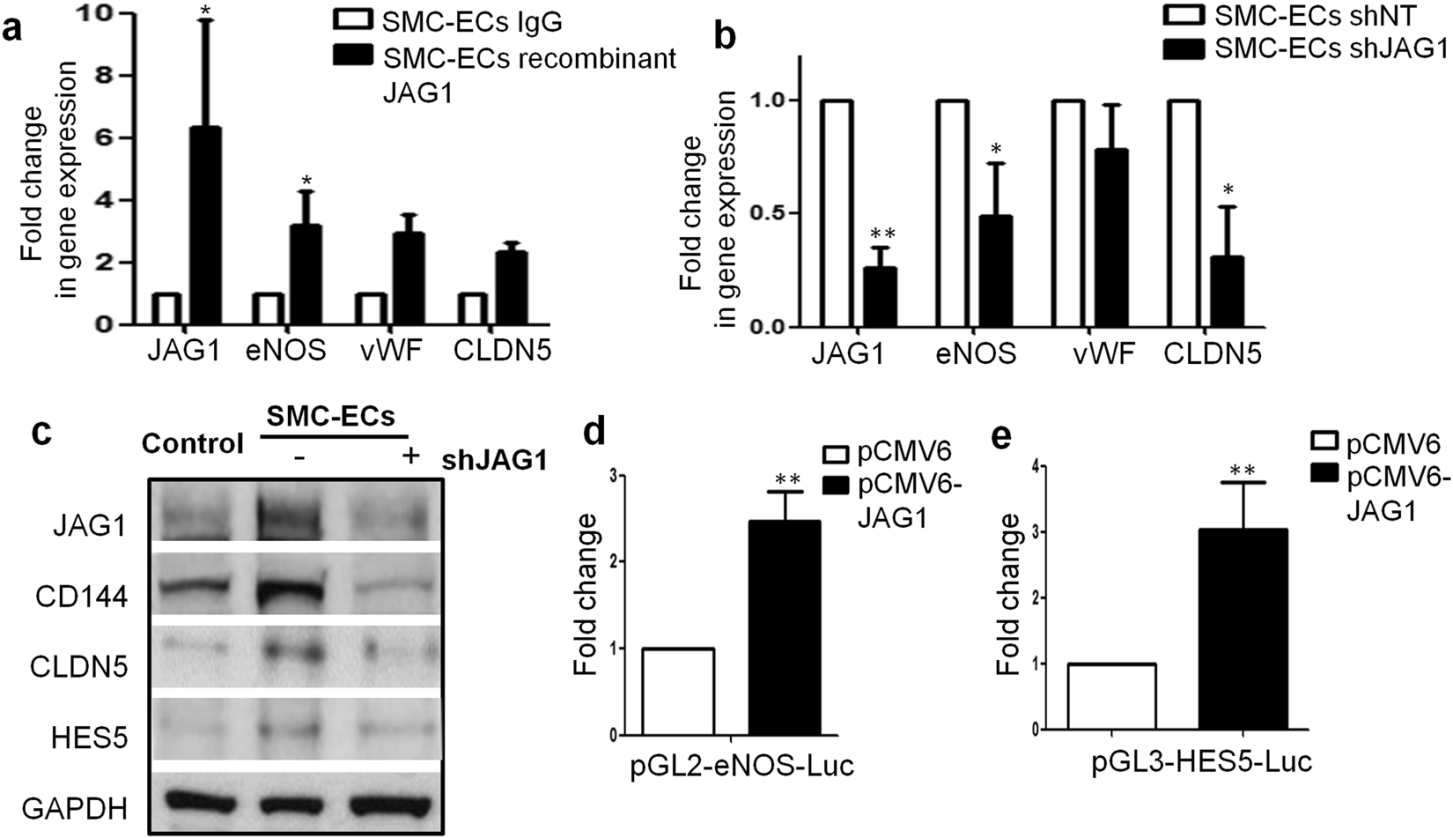
HES5 and JAG1 are implicated in endothelial lineage generation from PR-SMCs (II). (**a**) Real-time PCR revealed that after stimulating the cells with immobilized recombinant JAG1 from day 3 during differentiation, eNOS, vWF and Claudin-5 could be further upregulated (*p < 0.05 by Student’s *t* test, n = 3). (**b**) Knockdown JAG1 expression led to the downregulation of eNOS and Claudin-5 (*p < 0.05 by Student’s *t* test, n = 3). (**c**) Western blot analysis confirmed the downregulation of endothelial markers in concert with the JAG1 suppression. Control group refers to the SMC transfected with empty lentiviral vector that underwent the same reprogramming and differentiation protocol. Bands were cropped for the clarity. (**d**,**e**) Luciferase assay showed the activation of eNOS and HES5 promoter area with the transient overexpression of JAG1 (**p < 0.01 by Student’s *t* test, n = 3).

JAG1 is the upstream ligand of the Notch pathway and serves a much more complicated context-dependent role in regulating vascular activities^37, 38^. Upon recombinant JAG1 stimulation during differentiation, part of the endothelial markers, especially eNOS, could be induced to a higher level (Fig. 7a). Suppression of JAG1 through shJAG1 delivery caused expression impairment of the endothelial markers of the similar group (Fig. 7b,c). Interestingly, previous studies showed that JAG1 stimulation could activate eNOS in the presence of VEGF^38^. In our system, we observed that eNOS promoter was activated by JAG1 overexpression (Fig. 7d). Taken together, both HES5 and JAG1 participated in regulating endothelial differentiation. Furthermore, JAG1 knockdown led to the suppression of HES5 expression (Fig. 7c) and JAG1 overexpression could activate HES5 promoter (Fig. 7e). Above results suggested that JAG1 might carry out its endothelial regulatory function by acting on the downstream effector HES5. In addition, JAG1 activation was observed during SMC to PR-SMC dedifferentiation (Supplementary Fig. 14a-c). These results implied a wider regulatory function of JAG1 during both SMC dedifferentiation and endothelial differentiation. Taken together, our study focused on revealing the contribution of HES5 and JAG1, as a specific part of the Notch signalling involved in promoting EC differentiation.

## Discussion

In this study, we demonstrated for the first time the feasibility of the conversion of human SMCs to the endothelial lineage. The conversion was achieved using a two steps protocol consisting first of dedifferentiating the SMCs to CD34 positive vascular progenitors and subsequently differentiating the cells towards the endothelial lineage. Importantly, the SMC-derived ECs demonstrated angiogenesis capacity, especially in the animal ischemic disease model, and the ability to assemble into an endothelium-like layer in a tissue engineered vascular graft. Furthermore, our data revealed that the MET and the Notch signalling pathway were important mediators to obtain the cell fate conversion. Our findings are the first implication that human SMCs could represent a new valuable source of functional ECs for cell-based endothelial regeneration strategies.

IPS cells generating transcription factors *OCT4*, *SOX2*, *KLF4* and *c-MYC*, were used in this study to stimulate fully lineage committed SMCs back to an intermediate plastic progenitor state. The reprogramming time was set as four days which is much shorter than the time to generate full iPS cells in order to avoid the induction of pluripotency^25, 26^. After four days of reprogramming, the cells exhibited morphology changes and more importantly, an upregulation of the expression of CD34. CD34 expression has been associated with vascular progenitor cells which have the potential to give rise to both EC and SMC lineage in response to different conditions^9, 13^. It’s noteworthy that CD34 has also been used as a marker for circulating endothelial progenitor cells with the capacity to facilitate endothelial regeneration^28, 39^. The emergence of a CD34 positive population after our first step of reprogramming suggested the induction of a vascular progenitor state from SMCs. Interestingly, previous studies showed that the same set of reprogramming factors or *Oct4* alone could induce an upregulation in CD34 expression in fibroblasts in different culture conditions^13, 40^. Taken together, it appears that the short-term reprogramming step facilitates the erasing of the SMC signature and dedifferentiates the SMCs back to a progenitor state expressing CD34.

Accumulating evidence has shown that MET is a vital event at the initial stage of reprogramming for successful iPS cell generation^33–35^. Reprogramming factors coordinate a comprehensive molecular network which leads to the occurrence of MET and the reversion of the differentiated cells back to pluripotency. Furthermore, the participation of MET in the early stage of direct cell lineage conversions has been verified by recent studies converting fibroblast into embryonic Sertoli-like cells or cardiomyocytes^41, 42^. Collectively, MET is a key process involved in the manipulation of cell fate plasticity. SMC can be considered as mesenchymal-like cell due to the lack of intercellular adhesion, junction contacts and the ability to migrate through extracellular matrix (ECM). During the four days of reprogramming when the SMCs reversed back to vascular progenitors, we observed a downregulation of mesenchymal markers and an upregulation of cell-cell adhesion markers, especially E-Cadherin which is the characteristic event of MET. Moreover, inhibition of E-Cadherin impaired CD34 positive population induction from SMCs and abolished further endothelial differentiation. Above results revealed that MET is important for successful vascular progenitor generation from SMCs.

Following reprogramming, partially reprogrammed-SMCs (PR-SMCs) were subjected to endothelial-inductive conditions. We chose the combination of EGM-2 media and VEGF supplement to create an endothelial-favoured circumstance, which is based on the established protocols used for differentiating progenitors or pluripotent cells towards the endothelial lineage^12, 29^. After six days of stimulation, a portion of the total population converted to endothelial-like cells with morphology changes and the expression of endothelial markers at the mRNA and the protein level. These results proved the feasibility of the direct conversion of SMCs to ECs. To further improve the purity of derived endothelial-like cells for following functional tests, CD34 positive cells were selected from the whole PR-SMCs population. Indeed, CD34 positive PR-SMCs exhibited an enhanced capacity to give rise to a more enriched endothelial-like population with a complete repertoire of endothelial features. Taken together, we presented here a short and efficient protocol allowing the conversion of SMCs into ECs.

Notch signalling pathway widely participates in regulating diverse vascular cell activities during embryonic and postnatal development^43^. Comprehensive analysis of RNA-Seq data demonstrated the likely involvement of several Notch components in the SMCs to ECs conversion. Further analysis of the mRNA and protein expression profiles during the differentiation process led our interests particularly onto HES5 and JAG1. HES5 is a common downstream target of the Notch pathway which belongs to the basic helix-loop-helix transcription factors family and is usually associated with neural cell differentiation^44^. Recent reports suggested the role of HES5 in cardiovascular development as it might be a key positive mediator for the differentiation of bone marrow stromal cell into ECs^45^. HES5 has also been implicated in endothelial proliferation in response to endothelial injury during atherosclerosis^46^. In our protocol, HES5 was upregulated in concert with EC markers induction. Moreover, by knocking down or overexpressing HES5 during EC differentiation, we observed a repression or increase of EC markers expression respectively. All these results indicated the positive regulatory function of HES5 during endothelial induction. It’s possible that the four reprogramming factors open up certain opportunities for HES5’s further regulation on the endothelial related gene expressions.

JAG1, as the upstream ligand of the transmembrane receptors in the Notch pathway, plays a complicated role in orchestrating cell fate. Some studies provided the evidence of JAG1 facilitating endothelial differentiation and angiogenesis^37, 47^. Nevertheless, there were reports of the opposite effect in other cell models^48^. It is conceivable that in different cell types plus in response to different environmental cues, JAG1-activated Notch pathway may delicately control distinct regulation networks which lead to altered consequences. In this study, we revealed that JAG1 participated in the regulation of the expression of some EC markers especially eNOS during vascular progenitor to EC differentiation. We detected that upon JAG1 stimulation, eNOS was upregulated at the mRNA level through activation of its promoter. This finding coincided with a recent report that showed that the JAG1 activation in response to VEGF stimulation is required for eNOS activation and NO production in ECs^38^.

Cell-based therapeutic angiogenesis is a recently arising approach to restore blood perfusion to ischemic tissue. Successful therapeutic angiogenesis depends on the transplanted cells to directly incorporate into the neovasculature as well as to secrete angiogenic growth factors^2^. This therapy is of special significance in treating PAD since current pharmacological and interventional revascularisation therapies are not beneficial enough^49^. However, researchers are still investigating the optimal starting cells to generate functional angiogenic cells. In a murine hindlimb ischemia model of PAD for this study, we demonstrated that SMC-derived ECs could efficiently engraft into local vasculogenesis of ischemic tissue and profoundly improve the tissue perfusion. Moreover, to directly convert SMCs to ECs without reversing to a pluripotent state prevents the risk of tumour formation. Our results indicate that SMC-derived ECs could be a promising candidate for vascular regenerative therapies.

Tissue engineered vascular graft represents another promising direction of vascular regenerative medicine. In addition to direct transplantation to replace injured vessels, tissue engineered graft can also serve as an *ex vivo* useful model to study the physiology and pathophysiology related to vascular cells or ECM. Based on previous published protocol from our laboratory, functional vascular-resembling conduit can be generated from seeding the decellularized mouse aorta with human origin cells using an *ex vivo* bioreactor circulation system^30, 50^. Adopting this model, seeding the lumen of the decellularized vascular grafts with SMC-derived ECs led to successful reendothelialization which suggested the potential of those cells to construct tissue engineered vascular grafts. More importantly, our method provides the potential to construct tissue-engineered vascular graft with cells from the same origin. Based on our current result, the *in vivo* function of the reconstructed vascular graft could be further evaluated.

The proliferation and accumulation of SMCs following endothelial denudation/dysfunction profoundly contribute to the development of atherosclerosis and restenosis^4^. Our protocol proved the feasibility of direct conversion of SMCs into functional ECs *in vitro*, which provide a prospect for the further development of *in situ* endothelial regeneration therapy. To use the iPS cell-generating reprogramming factors as a first step of the protocol provide the cells with a clean slate for the further induction of endothelial phenotype. However, this approach still raises potential tumourigenesis risk^51, 52^. Although teratoma formation has not been observed in the *in vivo* experiments of this study, the long term effect is difficult to predict. Based on the feasibility of the conversion of SMC to an endothelial lineage and the possible underlying mechanisms we have discussed in this study, future work can explore the possibility of using other small molecules to partly or fully replace the reprogramming factors to reduce the safety concerns.

In summary, this is the first study proving that human vascular SMCs can be efficiently converted into functional endothelial lineage through an intermediate CD34 positive vascular progenitor state induced by the four reprogramming factors *OCT4*, *SOX2*, *KLF4* and *c-MYC*. By using *in vivo* and *ex vivo* models, we demonstrated the SMC-derived ECs have the potentials for therapeutic angiogenesis and constructing tissue-engineered vascular graft. We also provided insights into the mechanisms behind the process involving MET and Notch signalling. Further endeavours will focus on improving the efficiency of the current protocol with the use of small molecules or chemically defined conditions, as well as exploiting *in vivo* applications with eventual clinical benefits.

## Materials and Methods

### Cell culture

Human umbilical artery smooth muscle cells (UASMCs) (CC-2579, Lonza) were cultured on 0.05% Gelatin (from 2% Gelatin Solution in PBS, both from SIGMA) coated flasks in Smooth Muscle Cell Basal Media (SmBM, CC-3181, Lonza) supplemented with SmBM plus SingleQuots of Growth Supplements (CC-3182, Lonza) and maintained in a humidified incubator with 5% CO2 at 37 °C. Cells were passaged every other day at a ratio of 1:2. Cells used in this study were below passage 10. Human umbilical vein endothelial cells (HUVECs) were cultured on 0.5% Gelatin coated flasks in M199 medium (Gibco) supplemented with 10% fetal bovine serum and 1 ml EC supplement cocktail (contains 1ng/ml ECGF, 3 μg/ml EC growth supplement, 10unit/ml heparin, 1.25 μg/ml thymidine; all from Sigma-Aldrich). HEK 293 T (ATCC, CRL-11268) cells were cultured in DMEM media with 10% FBS in a humidified incubator with 5% CO2 at 37 °C. 293 T cells were passaged every other day at a ratio of 1:4.

### Cell dedifferentiation to PR-SMCs

SMCs were transfected with lentiviral vector encoding reprogramming factors Oct4, Sox2, Klf4 and c-Myc (TetO-FUW-OSKM, Addgene Plasmid 20321) or control empty vector and maintained in reprogramming media consisting of Knockout DMEM/F12 (12660-012, Invitrogen), 20% Knockout Serum Replacement (10828-028, Invitrogen), 0.1 mM β-mercaptoethanol, 0.1 mM MEM Non-Essential Amino Acids (Invitrogen) and 10 ng/ml basic Fibroblast Growth Factor (Miltenyi Biotec) on 0.05% Gelatin for 4 days. Media was changed every other day.

Alternatively, the four reprogramming factors were overexpressed using plasmid pCAG2LMKOSimO (Addgene, Plasmid 20866). The control empty plasmid was generated by removing the four gene-encoding ORF region. pCAG2LMKOSimO plasmid was first linearised by PvuI restriction enzyme (New England BioLabs) at site 12413. After linearisation, plasmid was purified with SureClean kit (Bioline). Transfection of SMCs with pCAG2LMKOSimO or control plasmid was performed using Basic SMC Nucleofector Kit (Lonza) as specified by manufacturer. Cells were then maintained in reprogramming condition same as described above for 4 days.

### Cell differentiation towards the endothelial lineage

PR-SMCs were transferred to Collagen IV (5 μg/ml in PBS, 354233, BD) coated flasks and maintained in EGM-2 Media (Endothelial Cell Growth Media Kit, CC-3162, Lonza) supplemented with 25 ng/ml VEGF (Recombinant Human VEGF165, 293-VE-010, Lonza) for 6 days to induce endothelial differentiation. Media was changed every other day.

### Cell sorting and culture of CD34 positive PR-SMCs

CD34 MicroBead Kit (Miltenyi Biotec) was used to select CD34 positive cells from PR-SMCs according to the manufacturer’s protocol. In brief, PR-SMCs were washed, trypsinized, counted and pelleted. The cell pellet was resuspended in resuspension buffer with 100 μl of FcR Blocking Reagent and 100 μl CD34 MicroBeads were added. Cell suspension was mixed well and incubated for 30 minutes. After incubation, cells were washed, pelleted and resuspended in 500 μl of buffer. Cell suspension was applied to a MS MACS separation column. Selected CD34 positive cells were grown in T25 flask coated with Collagen IV and cultured in EGM-2 media supplemented with 25 ng/ml VEGF.

### RNA extraction and real-time PCR analysis

Total cellular RNA was extracted with RNeasy Mini Kit (Qiagen) following the protocol provided by the manufacturer. 1 μg of total RNA was used to synthesize cDNAs using QuantiTect Reverse Trasncription Kit (Qiagen) according to the manufacturer’s specifications. Real-time PCR was performed using the SYBR Green PCR Master Mix (Applied Biosystems). ABI Prism 7000 Sequence Detection System instrument (Applied Biosystems) was used to carry out the PCR reaction. cDNA samples were denatured at 95 °C for 2 minutes, followed by 40 cycles amplification at 95 °C for 15 seconds and 60 °C for 1 minute and finally extended at 72 °C for 1 minute. Threshold cycle numbers (Ct) were measured in the exponential phase for all samples. Fold changes were calculated as the relative fold difference of Ct value of target gene against GAPDH housekeeping gene. The primers used for real-time PCR experiments are listed in Table S1.

### Western Immunoblotting

Whole cell lysate protein was extracted by using RIPA Lysis Buffer (Santa Cruz) and sonicated with Branson Snifier 150. The concentration of extracted protein was measured based on Bradford dye-binding method by using Bio-Rad Protein Assay Reagent and Bio-Rad SmartSpecTM 3000 spectrophotometer. Protein samples were fractionated by size on NuPAGE 4–12% Bis-Tris Gel (Novex) by electrophoresis and then transferred to Nitrocellulose Membrane (Protran, Whatman). The probed primary antibodies were detected by using Horseradish Peroxidise (HRP)-conjugated secondary antibodies and the Enhanced Chemiluminescent (ECL) detection system (GE Health). Primary antibodies: α-SMA (A5228, Sigma-Aldrich, 1:1000); SM22α (ab14106, Abcam, 1:1000); Calponin (EP798Y, Abcam, 1:1000); FSP-1 (ab27957, Abcam, 1:500); SOX2 (ab59776, Abcam, 1:500); OCT4 (sc-5279, Santa Cruz, 1:500); KLF4 (sc-20691, Santa Cruz, 1:500); CD144 (ab33168, Abcam, 1:500); CD31 (252253, ABBIOTEC, 1:500); Claudin 5 (352588, life technologies, 1:500); E-Cadherin (3195 P, Cell Signaling, 1:500); SNAI1 (3879 P, Cell Signaling, 1:1000); N-Cadherin (ab12221, Abcam, 1:300); HES5 (Ab5708, Millipore, 1:400); JAG1 (sc8303, Santa Cruz, 1:300); GAPDH (ab8245, Abcam, 1:2000). Secondary antibodies: anti-rabbit (P0217, DAKO, 1:3000); anti-mouse (P0260, DAKO, 1:3000).

### Immunofluorescence staining

Cells were seeded on glass slide and cultured in corresponding media until achieved 80% confluency. After washing with PBS, cells or frozen sections were fixed with 4% paraformaldehyde (PFA) (Sigma-Aldrich) for 15 minutes and then permeabilized with 0.1% Triton X-100 (Sigma-Aldrich) for another 15 minutes. Samples were blocked with 5% appropriate serum/1 × PBS at 37 °C for 30 minutes followed by incubation with primary antibodies at 37 °C for 1 hour: CD34 (sc7324, Santa Cruz, 1:50); CD31 (sc1506, Santa Cruz, 1:50); eNOS (610297, BD Biosciences, 1:50); CD144 (sc6458, Santa Cruz, 1:50); KDR (ab9530, Abcam, 1:100); human specific CD31 (ab32457, Abcam, 1:100); HES5 (Ab5708, Millipore, 1:50); vWF (ab6994, Abcam, 1:100); VCAM-1 (sc-13160, Santa Cruz, 1:50); ICAM-1 (MA5-13021, ThermoFisher, 1:50). After washing with PBS, samples were incubated with the appropriate Alexa Fluor-conjugated secondary antibodies (Invitrogen, 1:500) for 45 minutes at 37 °C. Samples were washed with PBS and then DAPI (Sigma Aldrich) was applied for 3 minutes. The slides were mounted with Fluorescent Mounting Media (Dako) and images were acquired by Olympus IX81 microscope equipped with TRITC, FITC and DAPI filters.

### Flow cytometry analysis

Flow cytometry was performed to analyze the percentage of endothelial markers expression in different cell samples. Cells were harvested, washed with ice-cold FACS buffer (PBS supplemented with 2% FBS and 5 mM EDTA) and incubated with conjugated antibody for 30 minutes on ice. Compensation was performed with CompBeads (BD). Specific fluorochrome-conjugated antibodies were applied to stain the cell surface: CD34-PerCP (343519, Biolegend); CD34-FITC (343504, Biolegend); CD31-PE (303106, Biolegend); CD309(VEGFR2/KDR)-PE (359903, Biolegend); CD117(C-KIT)-PE (313203, Biolegend); CD140a(PEGFRα)-PE (323505, Biolegend); SSEA4-PE (330405, Biolegend). BD Accuri C6 Cytometer was used to perform flow cytometry. Data was collected and analyzed with FlowJo V10. For double staining analysis, fluorescence-minus-one (FMO) analyses were performed on Control or PR-SMCs to set the threshold for CD34 and CD31/KDR/C-KIT/PDGFRα positivity.

### RNA-Sequencing Analysis

The following groups were analyzed: UASMCs, HUVECs, PR-SMCs, SMC-ECs and SMC-CD34-ECs. Total RNA was extracted using RNeasy Mini Kit (Qiagen) following the manufacturer’s protocol. RNA quantity and quality were checked with NanoDrop ND-1000 spectrophotometer (NanoDrop Technologies, Wilmington, DE) and electrophoresis on a denaturing agarose gel. cDNA libraries were prepared using Illumina TruSeq RNA-Seq sample preparation kit (Illumina, San Diego, CA). The libraries were analyzed using Illumina HiSeq2500 (Illumina, CA) by GENEWIZ (NJ, USA). Results were generated as fastq files and sequence reads were trimmed to remove low quality bases at ends. The raw RNA-seq data are deposited to SRA as BioSample accessions: SAMN06309997, SAMN06309998, SAMN06309999, SAMN06310000, SAMN06310001. Then sequence reads were mapped to the reference of Homo sapiens genome using CLC Genomics Server program. Hit counts and reads per kilobase per million (RPKM) values were calculated for genes, which is first to align the raw read counts to exons of mRNA transcripts in RefSeq and then normalised these values to total uniquely aligning reads and transcript length.

Gene expression analysis was based on the normalized RPKM values of genes, including global gene expression analysis and expression comparisons for individual genes of interest. Differential expression was defined as a minimum 2 folds change. Global gene expression analysis was performed with Cluster 3.0 with average linkage. Genes with the RPKM values below 0.5 were removed from the analysis. The results were visualized with Java TreeView in the form of red-green heat map. Gene Ontology (GO) analysis was performed on the upregulated genes in SMC-CD34-ECs compared to SMCs. DAVID bioinformatics resource 6.7 functional annotation tool suite was used to carry out the GO analysis. P-value is calculated by the software and represents the significance of the particular GO term associated with the group of genes.

### Acetylated LDL uptake assay

70% confluent SMC-ECs or empty vector transfected control cells on slide were incubated with 10 μg/ml Alexa Fluor 594 conjugated Ac-LDL (L-35353, life technologies) for 4 hours in culture media at 37 °C. Cells were then washed with PBS and stained with DAPI. Samples were immediately visualized under fluorescent microscope.

### Adhesion molecule expression in response to TNFα stimulation

SMCs, SMC-ECs, and HUVECs were seeded and grown in 4-well chamber slides. They were stimulated with 10ng/ml TNFα for 6 hours before immunofluorescence stained with ICAM-1 antibody (MA5-13021, ThermoFisher Scientific).

### *In vitro* Matrigel Tube Formation Assay

100 μl Matrigel (BD Biosciences) was added to each chamber of an 8-chamber slide and allowed to solidify for 1 hour at 37 °C. 1 × 10^5^ cells suspended in 300 μl culture media were then added into each Matrigel-coated chamber. Chamber slide was maintained in 37 °C incubator. Tube formations were observed at 6 hours time point with light microscope and images were taken. Total tube length was quantified with ImageJ image processing program. Immunofluorescence staining was then performed similarly to cell staining described above but with prolonged incubation times.

### *In vivo* Matrigel Plug Assay

NOD.CB17-*Prkdc* ^*scid*^/NcrCrl mice were anaesthetized with sodium pentobarbital (50 mg/kg body weight). 5 × 10^5^ SMC-CD34-ECs or control cells were mixed well with 50 μl EGM-2 media supplemented with 25 ng/ml VEGF and 250 μl Matrigel. The cell mixture was injected subcutaneously into the mice. Six injections were conducted for each group. The mice were sacrificed by cervical dislocation 14 days later and the plugs were harvested. Samples were fixed in liquid nitrogen, cryosections were prepared and immunofluorescence staining was then performed. To further confirm the involvement of injected cells, SMC-CD34-ECs were labelled with Molecular Probes Vybrant Dil Cell Labelling solution (life technologies) before injection.

### Mouse Hindlimb Ischemia Model

The ischemia model was performed as previously described^53^. NOD.CB17-*Prkdc* ^*scid*^/NcrCrl mice were anaesthetized with sodium pentobarbital (50 mg/kg body weight). The right femoral artery was ligated permanently. SMC-CD34-ECs, SMCs or PBS were injected intramuscularly into the adductors. The blood flow of ischemic hindlimb was evaluated by LDI Doppler laser scanner (Moor Instruments) 30 minutes post surgery and 2 weeks after. The blood flow ratio was defined as the ratio of mean measurement in the foot area of ligated hindlimb to the contralateral unligated hindlimb. The mice were sacrificed by cervical dislocation after flow measurement at 2 weeks and adductor muscular tissues of ligated side were harvested following cryosectioning and immunofluorescence staining with CD31 antibody for in general evaluation of capillary density and human specific CD31 antibody to evaluate the engraftment of injected human cells to neo-vascularization.

### Generation of dual seeding vessel graft using ***ex vivo*** bioreactor system

The experiment was performed as previously described^49, 54^. In brief, the mice were sacrificed by cervical dislocation and the thoracic aorta was excised from the mouse and immediately flushed with saline solution containing 100U heparin to prevent the formation of blood clots. Peri-aortic connective tissue was gently removed with forceps. The aorta lumen was flushed with 5 ml 0.075% sodium dodecyl sulphate (SDS) solution (Severn Biotech Ltd) diluted in PBS and then soaked in the same solution for 2 hours on an orbital shaker. Then the aorta was flushed and washed in PBS to finish the decellularization process. The decellularized aorta graft was then fixed in the incubation chamber of a special customized bioreactor circulation system (Zyoxel Ltd, Oxford, UK). The complete setup was maintained in a standard at 37 °C incubator. 1 × 10^6^ SMC-CD34-ECs were mixed with 50 μl of EGM-2 media and seeded inside the decellularized vessel graft via direct injection. 1 × 10^6^ SMCs were mixed with 100 μl of Matrigel and pipetted onto the graft to form a gel-like wrap. After 12 hours of static incubation to allow the attachment of the cells to the graft, EGM-2 media was delivered through the lumen by a peristaltic pump with initial flow rate at 5 ml/min followed by a stepwise increase to 20 ml/min over 24 hours. The flow rate was then kept at 20 ml/min for 5 days. The circulating and chamber medium were changed every other day.

### ShRNA lentiviral particles transduction

Lentiviral particles were generated using shRNA E-Cadherin (CDH1 MISSION shRNA, SHCLNG_NM_004360, Sigma Aldrich), shRNA HES5 (HES5 MISSION shRNA, SHCLNG_XM_371215, Sigma Aldrich) and shRNA JAG1 (JAG1 MISSION shRNA, SHCLNG_NM_000214, Sigma Aldrich) according to the protocol previously described 5. Briefly, the shRNA Non-Targeting (NT) vector was used as negative control. HEK 293 T cells were tranfected with the lentiviral vector and the packaging plasmids, pCMV-dR8.2 and pCMV-VSV-G (both from Addgene) using FuGENE HD (Promega). The supernatant containing the lentiviral particles was collected and filtered 48 hours after transfection. The viral tilters were determined using the p24 antigen ELISA (Zeptometrix). For lentiviral transduction, cells were seeded overnight and the following day the cells were incubated with shRNA or NT control (1 × 10^7^ Transducing Units/ml) in complete medium supplemented with 10 µg/ml of Polybrene for 16 hours. Subsequently fresh medium was added to the cells and the plates were harvested at the stated time points after transduction.

### HES5 overexpression by plasmid nucleofection

Plasmid pCMV6-HES5 (RG215311, Origene) containing full length human HES5 cDNA fragment was used to overexpress HES5 at day 2 of differentiation from PR-SMCs to SMC-ECs. Empty pCMV6 vector was used as negative control. Cells were transfected with pCMV6-HES5 or pCMV6 using Basic Nucleofector Kit (LONZA) as specified by manufacturer. The expression of the endothelial markers was evaluated at the transcriptional and protein levels after 72 hours of nucleofection.

### Luciferase activity assay

3 days differentiated PR-SMCs were transiently transfected with reporter plasmid pGL2-eNOS Promoter luciferase (Plasmid 19297, Addgene) containing 1621 bp fragment of human eNOS promoter and reporter plasmid Renilla (Promega) in the presence of pCMV6-JAG1 or pCMV6 empty vector (HG11648-M-N, Sino Biological) using Fugene HD (Promega). After 48 hours of incubation, cells were lysed in lysis buffer (Promega). Luciferase and renilla activities were measured with the dual Luciferase Assay System (Promega) with a luminometer (Lumat LB 9507, Berthold Technologies). Renilla activity was used to normalize the transfection efficiency. In another set of experiments, cells were transfected with pGL3-HES5 Promoter Luciferase and Renilla in the presence of pCMV6-JAG1or pCMV6 empty vector.

### Immobilized recombinant JAG1 stimulation

Six-well cell culture plates were coated with 50 μg/ml of Protein G (life technologies) in PBS at room temperature (RT) over night. Then the plates were washed 3 times with PBS and further blocked with 10 mg/ml BSA in PBS for 2 hours at RT. The plates were washed 3 times with PBS before incubation with 5 μg/ml of recombinant Jagged1-FC chimera (R&D systems) in 0.1% BSA/PBS or only IgG as control for 4 hours at RT. After washing 3 times with PBS, 3 days differentiated PR-SMCs were seeded on the coated plates and maintained in the same endothelial inducing conditions.

### Statistical Analysis

Statistical analyses were performed using the GraphPad Prism 5.0 software (GraphPad Software, Inc.). Data were analyzed by two-tailed Student’s *t* test or analysis of variance (ANOVA), when *t* test is inappropriate, followed by multiple comparisons with Bonferroni’s method. Data were presented as the mean and standard error of the mean (S.E.M.). A value of p < 0.05 was considered to be significant.

### Study Approval

All animal procedures were performed according to protocols approved by the Institutional Committee for Use and Care of Laboratory Animal and all the animal experiments were performed conform the guidelines from Directive 2010/63/EU of the European Parliament on the protection of animals used for scientific purposes.

## Electronic supplementary material

Supplementary figures and table
