# Supplementary material for: Transdifferentiated Human Vascular Smooth Muscle Cells are a New Potential Cell Source for Endothelial Regeneration
Source: Sci Rep. 2017 Jul 17;7:5590. doi: 10.1038/s41598-017-05665-7 (PMC5514066; doi:10.1038/s41598-017-05665-7)
Supplement: Supplementary file 1 — Supplementary figures and table [file 41598_2017_5665_MOESM1_ESM.pdf]

Transdifferentiated Human Vascular Smooth Muscle Cells are a New Potential Cell Source for Endothelial Regeneration

Xuechong Hong, PhD[1], Andriana Margariti, PhD[2], Alexandra Le Bras, PhD[1], Laureen Jacquet, PhD[1], Wei Kong, MD, PhD[3], Yanhua Hu, MD[1], and Qingbo Xu, MD, PhD[1*]

[1]Cardiovascular Division, BHF Centre for Vascular Regeneration, King's College London, London, UK

[2]Centre for Experimental Medicine, School of Medicine, Dentistry and Biomedical Sciences, Queen's University Belfast, Belfast, UK

[3]School of Basic Medical Sciences, Peking University; Key Laboratory of Molecular Cardiovascular Science, Ministry of Education, Beijing, China

*Correspondence should be addressed to Qingbo Xu, MD, PhD at Cardiovascular Division, King's College London BHF Centre, 125 Coldharbour Lane, SE5 9NU London, UK.

Email: qingbo.xu@kcl.ac.uk Tel: +44 (0)20 7848 5295 Fax: +44 (0)20 7848 5296

Supplementary Information

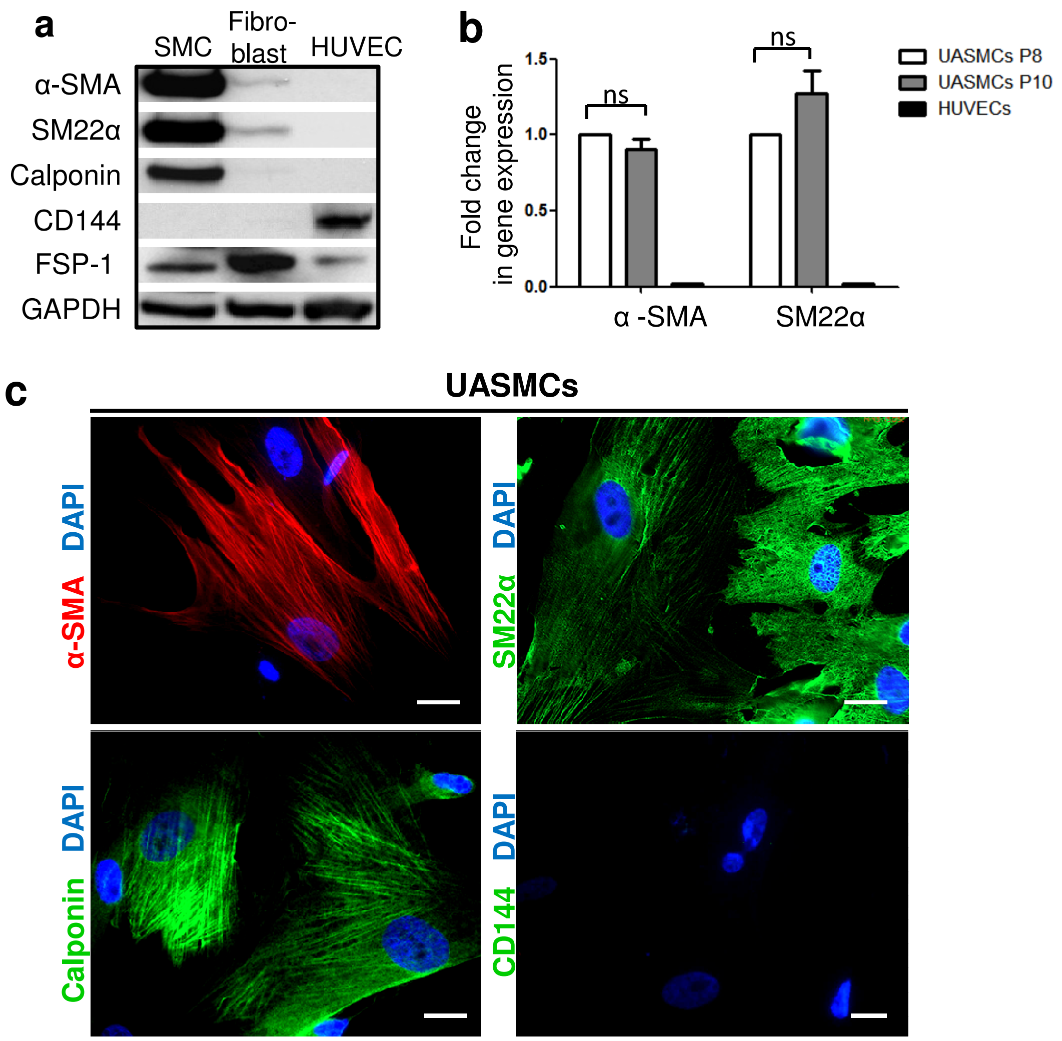

Supplementary Figure 1. Characterization of the human Umbilical Artery Smooth Muscle Cells (UASMCs). (a) Western blot analysis showed that human umbilical artery SMCs (UASMCs) strongly expressed SMC markers α-SMA, SM22α, Calponin, have no expression of endothelial marker CD144 and very low expression of FSP-1. Human fibroblasts and human umbilical vein endothelial cells (HUVECs) were used as controls. **(b)** Real-time PCR results showed UASMCs maintained SMC marker expression along cell passaging. (ns=non significant by Student's *t* test, n=3). **(c)** Representative images of the immunofluorescence staining of UASMCs for SMC markers α-SMA, SM22α, Calponin and endothelial marker VE-Cadherin. (Scale bar: 25μm)

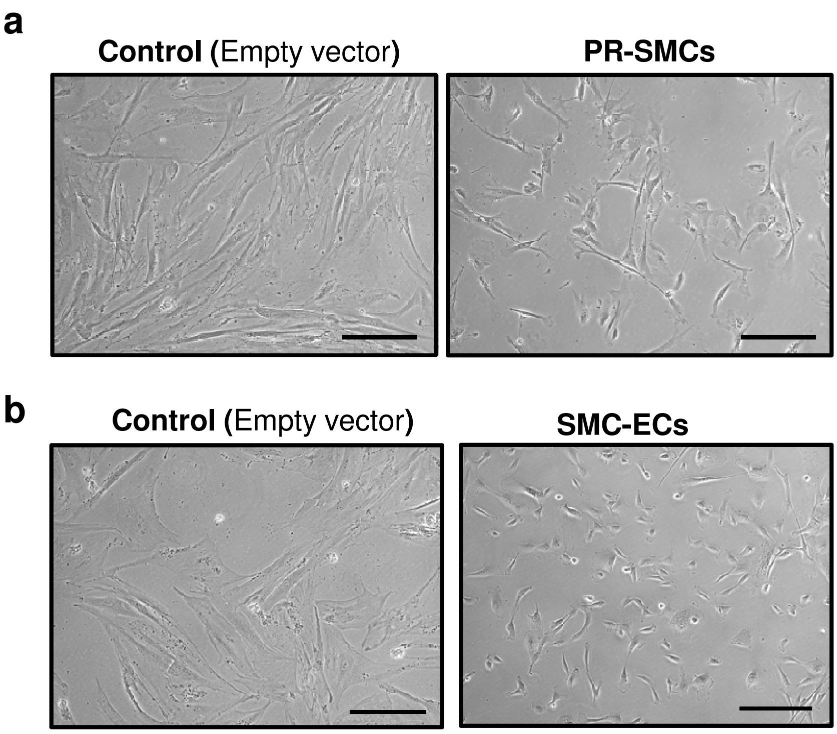

Supplementary Figure 2. Cell morphology of PR-SMCs and SMC-ECs. (a) Representative image of PR-SMCs displayed a distinct morphology compared to the control group. SMCs transfected with empty lentiviral vector and kept under identical reprogramming conditions were used as the control group. (Scale bar: 100μm) **(b)** SMCs converted ECs (SMC-ECs) exhibited profound morphology change compared to the control group. SMCs transfected with an empty lentiviral vector that underwent the same reprogramming and differentiation protocol were used as control group. (Scale Bar: 100μm).

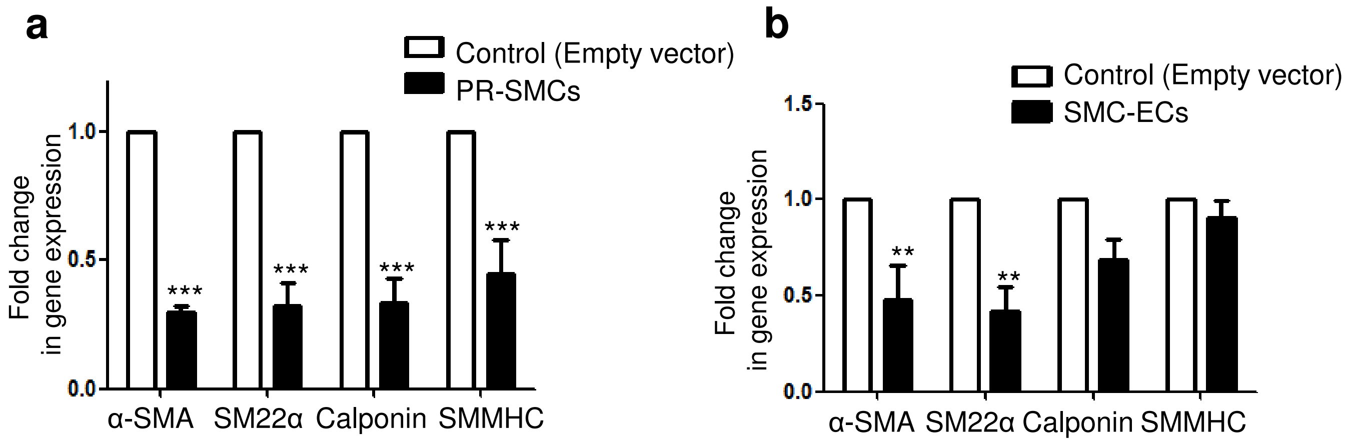

Supplementary Figure 3. The downregulation of SMC markers expression by PR-SMCs and SMC-ECs. (a) Real-time PCR analysis showed an overall suppression of typical SMC markers in PR-SMCs compared to the control cells. SMCs transfected with empty lentiviral vector and kept under identical reprogramming conditions were used as the control group. (***p<0.001 by Student's *t* test, n=3) **(b)** Real-time PCR analysis showed an overall suppression of typical SMC markers in SMC-ECs compared to the control cells. SMCs transfected with an empty lentiviral vector that underwent the same reprogramming and differentiation protocol were used as control group. (***p<0.001 by Student's *t* test, n=3).

Supplementary Figure 4. **Hong et al**

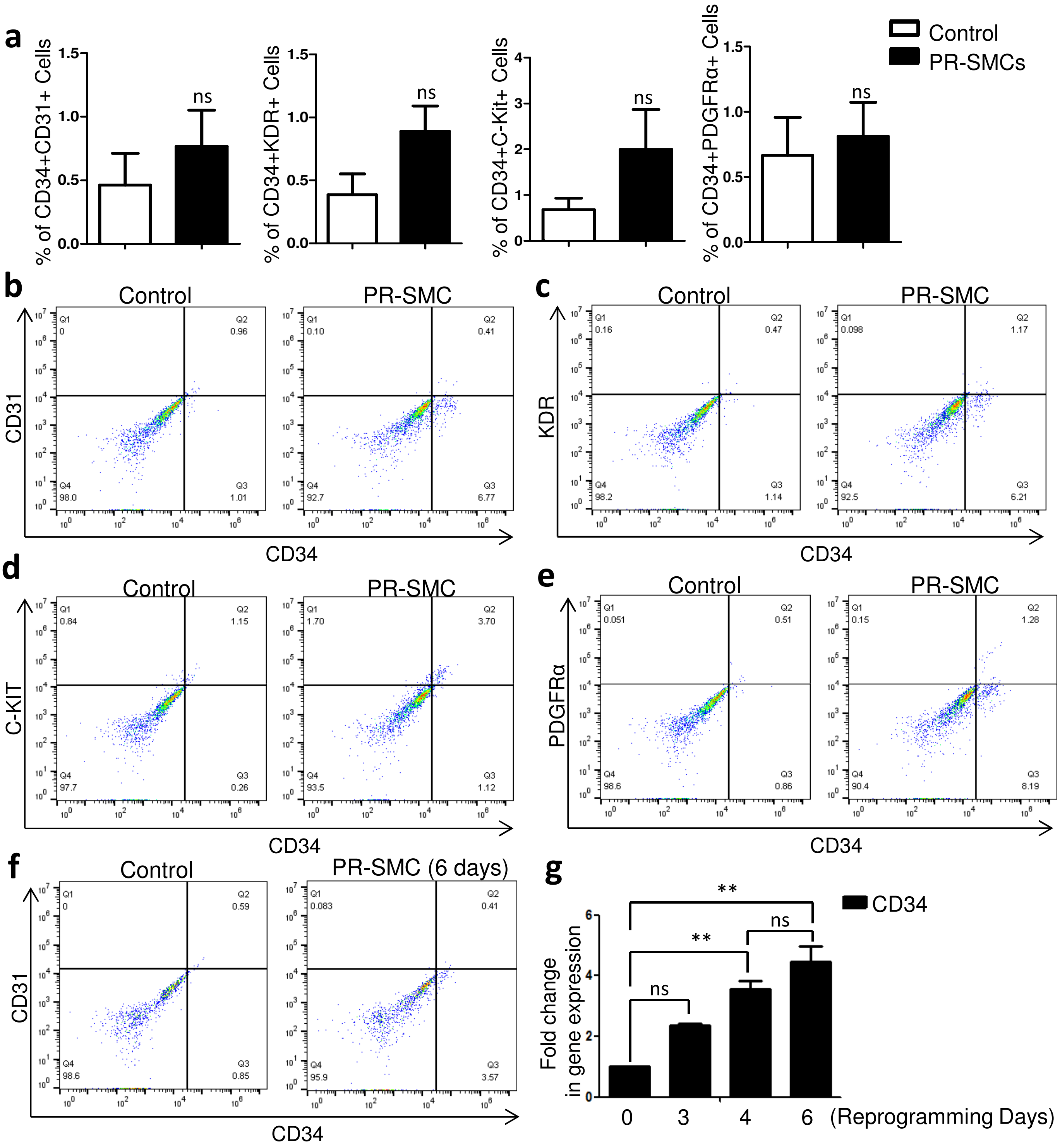

Supplementary Figure 4. Analysis of PR-SMCs phenotypic characteristics. (a) Flow cytometry analysis of CD34 with CD31, KDR, C-KIT and PDGFRα double positive populations in control or PR-SMCs. (ns=non significant by Student's *t* test, n=3) Representative flow cytometry analysis of **(b)** CD34/CD31, **(c)** CD34/KDR, **(d)** CD34/C-KIT and **(e)** CD34/PDGFRα double staining of PR-SMCs. **(f)** Flow cytometry analysis of CD34/CD31 double staining for SMCs reprogrammed with four reprogramming factors for 6 days. **(g)** CD34 expression at different time point during SMCs dedifferentiation was evaluated with real-time PCR.(**$p<0.01$, ***$p<0.001$ by one-way ANOVA followed by multiple comparisons with Bonferroni's method, n=3)

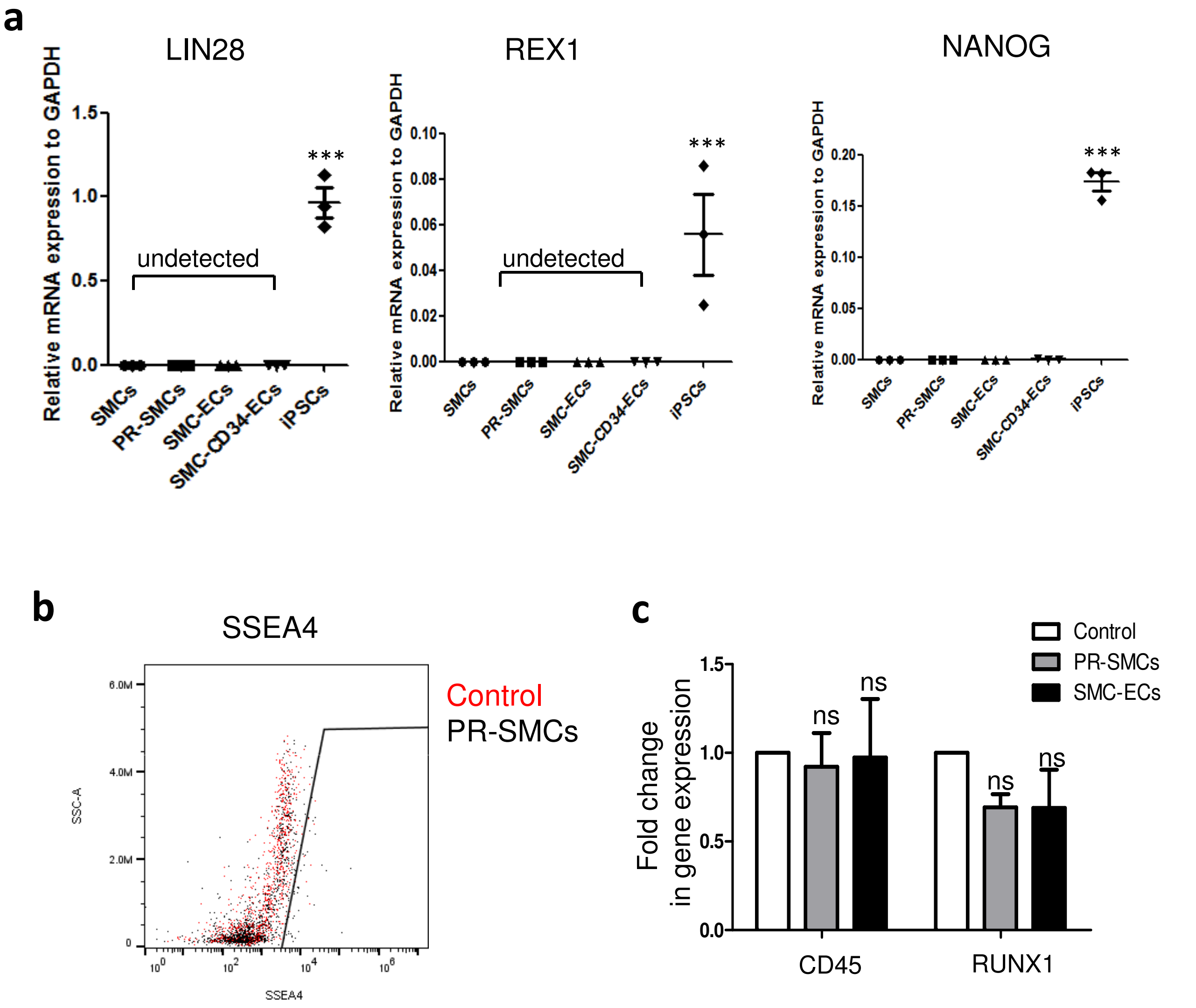

Supplementary Figure 5. Evaluation of pluripotency and hematopoietic markers expression along SMC to endothelial lineage conversion. (a) Real-time PCR analysis showed that compared with human iPS cells, none of the PR-SMCs, SMC-ECs and SMC-CD34-ECs population expressed comparable level of pluripotency markers LIN28, REX1 and NANOG. Primers that detected LIN28 and REX1 in human iPS cells could not detect LIN28 and REX1 expressions in PR-SMCs, SMC-ECs or SMC-CD34-ECs and the Ct values were manually set as 35 for undetected samples. **(b)** Flow cytometry analysis did not detect significant SSEA4 expression in the PR-SMCs population. **(c)** Real-time PCR analysis showed no significant change in hematopoietic progenitor marker CD45 or RUNX1 expression in PR-SMCs or SMC-ECs. (ns=non significant by Student's *t* test, n=3)

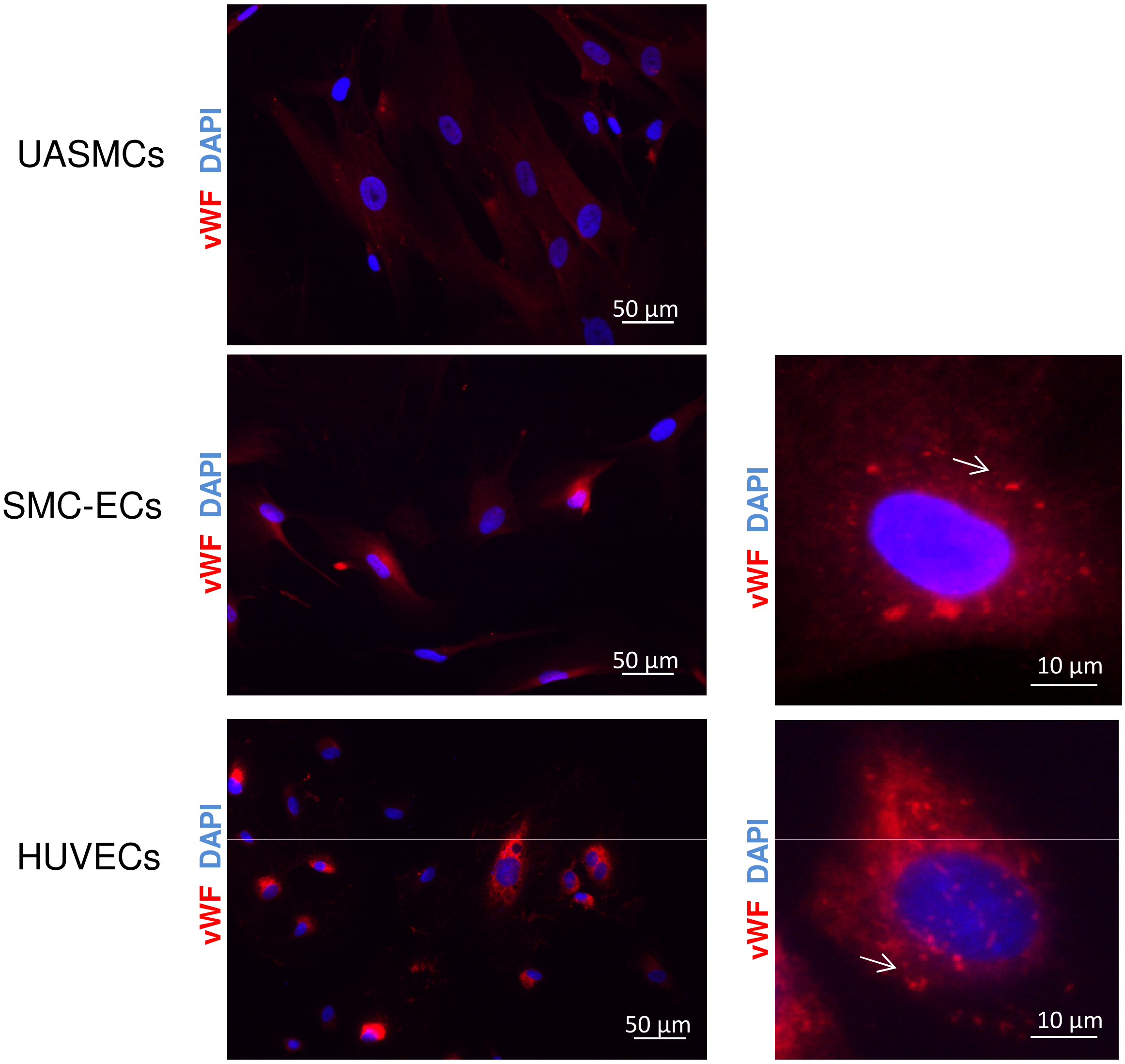

Supplementary Figure 6. Immunofluorescence staining of SMC-ECs for vWF. Representative images of the immunofluorescence staining for endothelial marker vWF of SMC-ECs. SMC-ECs stained with vWF showed around the nuclei staining pattern similar to HUVECs. White arrows in the enlarged images showed that vWF started to form Weibel-Palade body-like rod-shaped structures in the SMC-ECs.

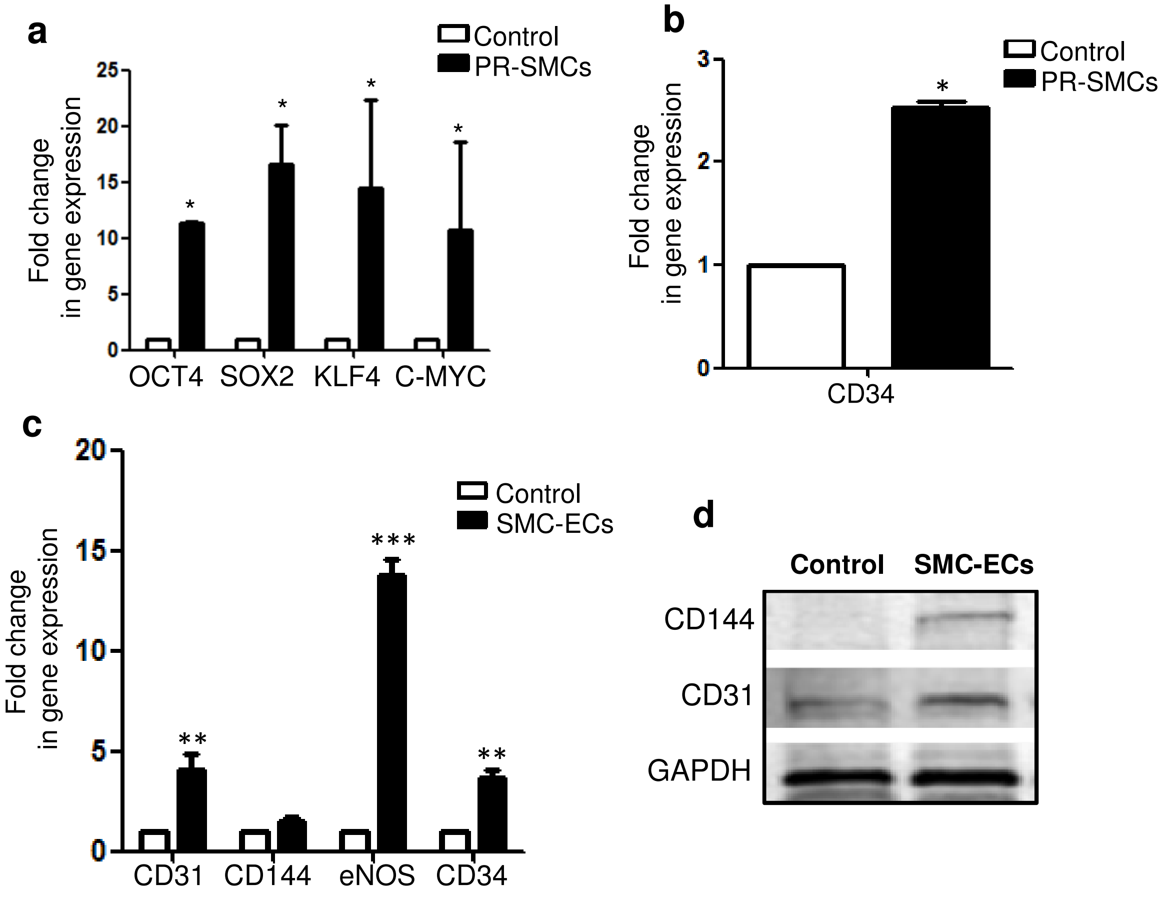

Supplementary Figure 7. Conversion of SMCs towards endothelial lineage using pCAG2LMKOSimO plasmid encoding the four reprogramming factors. (a) After 4 days reprogramming following pCAG2LMKOSimO plasmid or empty plasmid transfection, real-time PCR showed the overexpression of OCT4, SOX2, KLF4 and c-MYC in PR-SMCs compared to the empty vector control cells. (*p<0.05 by Student's *t* test, n=3) **(b)** Real-time PCR revealed the upregulation of CD34 of PR-SMCs. (*p<0.05 by Student's *t* test, n=3) **(c)** After 6 days of endothelial-inductive differentiation, endothelial markers CD31, eNOS, CD34 were upregulated in SMC-ECs at the mRNA level confirmed by real-time PCR. (**p<0.01, ***p<0001 by Student's *t* test, n=3) **(d)** Western blot analysis demonstrated the induction of CD144 and CD31 of SMC-ECs. In this figure, all "Control" refer to the SMCs that transfected with empty pCAG vector that underwent the same reprogramming and differentiation protocol.

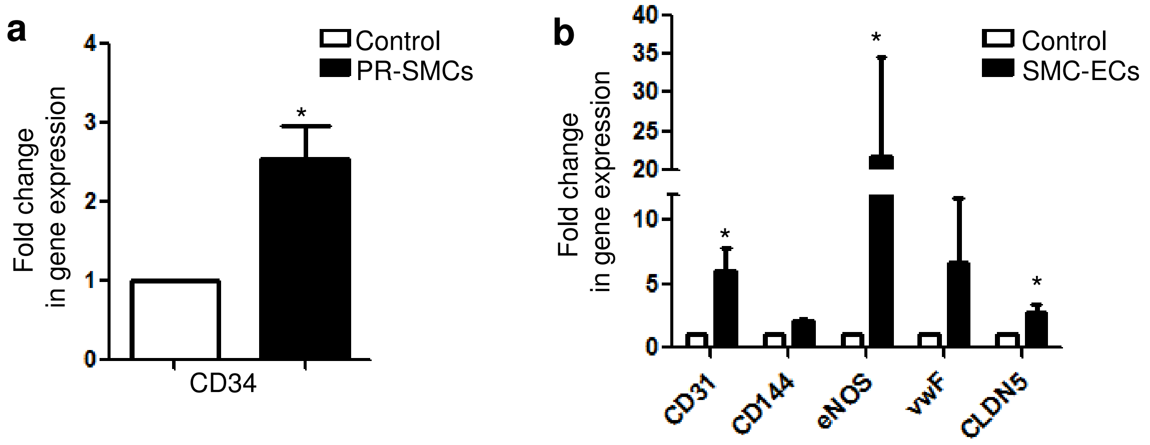

Supplementary Figure 8. Verification of the protocol with UASMC from another batch. (a) Real-time PCR revealed the upregulation of CD34 of PR-SMCs. (*$p<0.05$ by Student's *t* test, n=3) **(b)** After 6 days of endothelial-inductive differentiation, endothelial markers CD31, eNOS, CD34 were upregulated in SMC-ECs at the mRNA level confirmed by real-time PCR. (*$p<0.05$ by Student's *t* test, n=3)

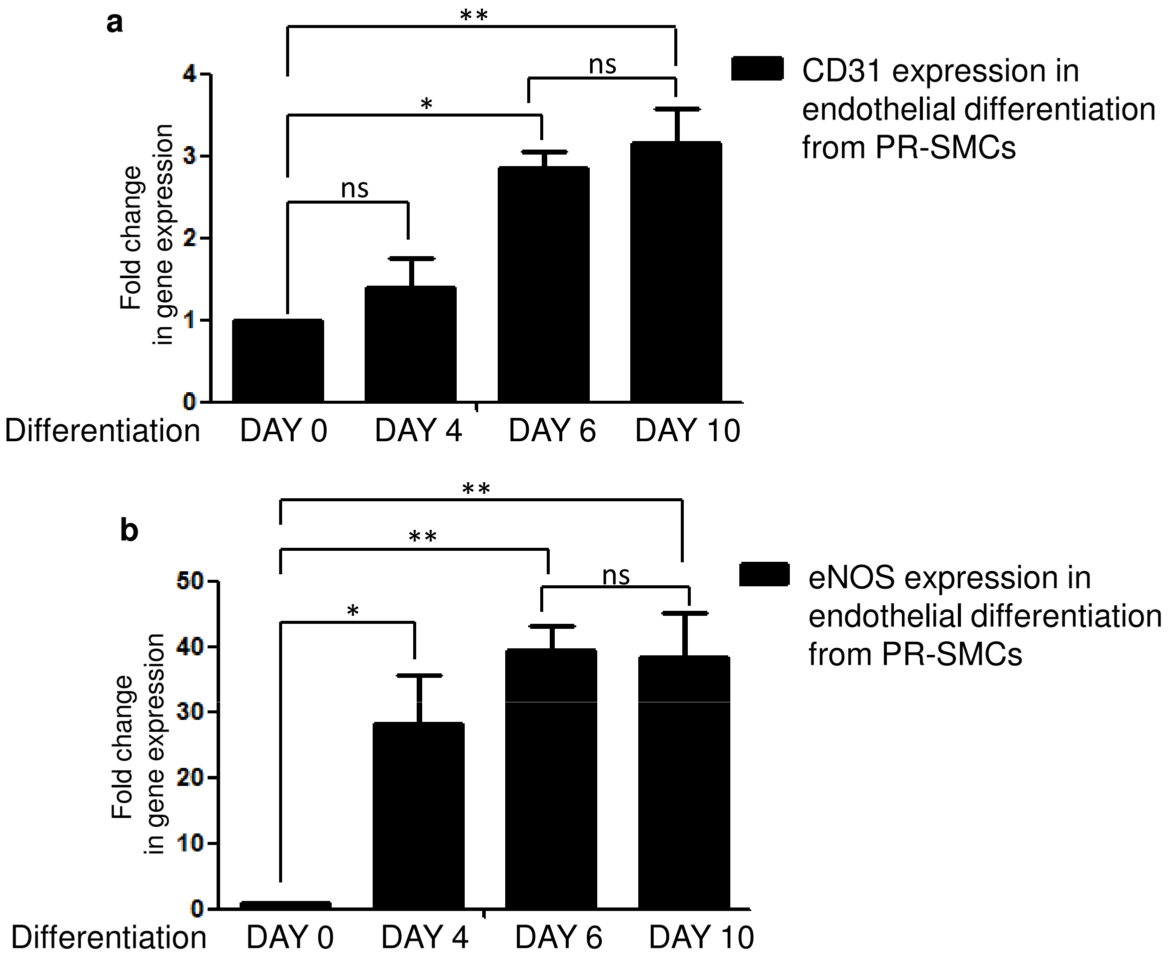

Supplementary Figure 9. Endothelial marker expression during PR-SMC to endothelial differentiation. Real-time PCR revealed the course of **(a)** CD31 and **(b)** eNOS upregulation during endothelial differentiation from PR-SMCs (ns=non significant, *p<0.05, **p<0.01 by Student's *t* test, n=3)

Gene Ontology Analysis
SMC-CD34-ECs vs UASMC

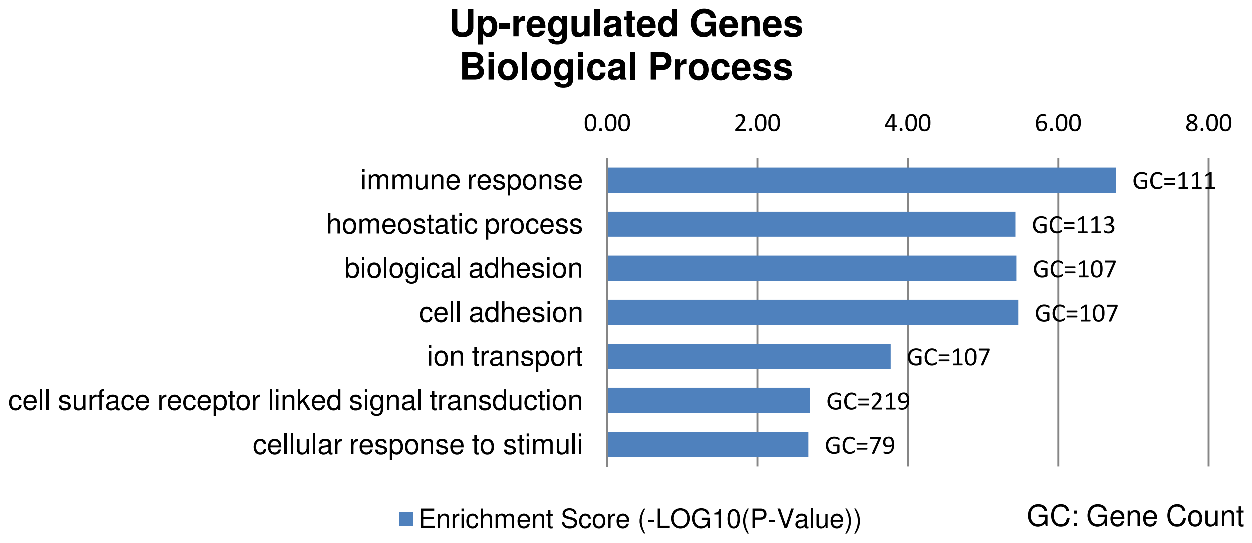

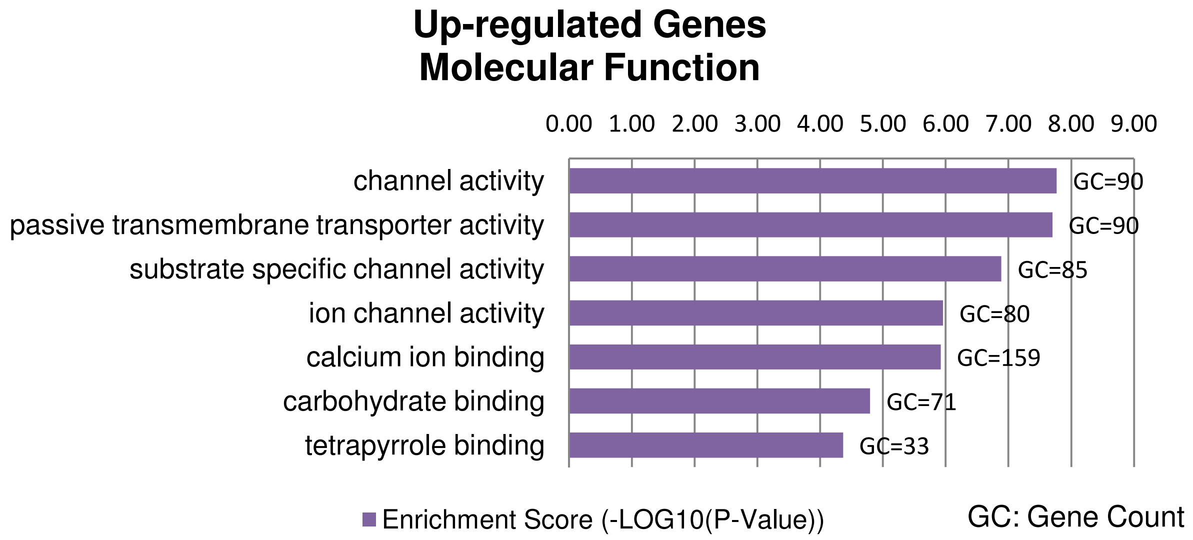

Supplementary Figure 10. Gene ontology analysis of the upregulated genes between SMC-CD34-ECs and SMCs. Gene ontology analysis was performed on the upregulated genes of SMC-CD34-ECs compared to SMCs from the RNA-Seq data. Enrichment in gene ontology of biological process and molecular function were revealed. Significance of enrichment was evaluated by p-value.

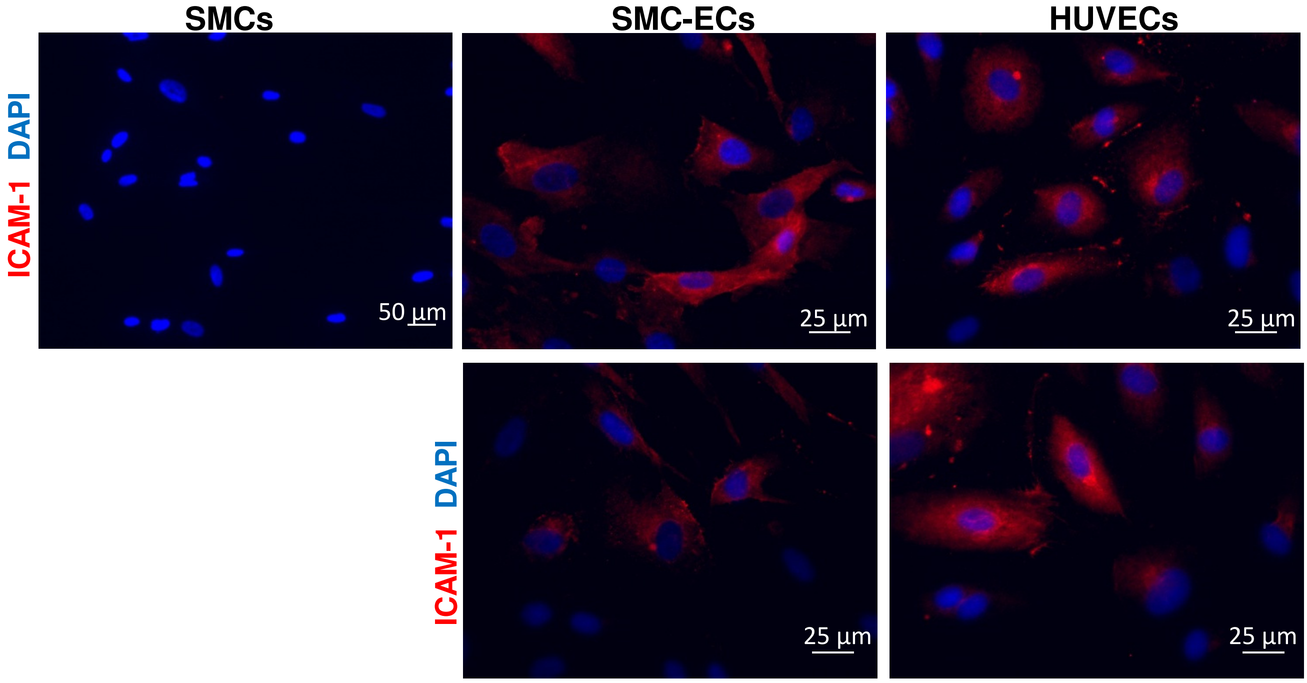

Supplementary Figure 11. Immunofluorescence staining of SMC-ECs for ICAM-1 in response to human TNFα stimulation. Representative images of the immunofluorescence staining for ICAM-1 expression in SMC-ECs in response to human TNFα stimulation. Data are from a single experiment (n=3) representative of 2 independent experiments. SMC-ECs stained with ICAM-1 showed a similar staining pattern with HUVECs.

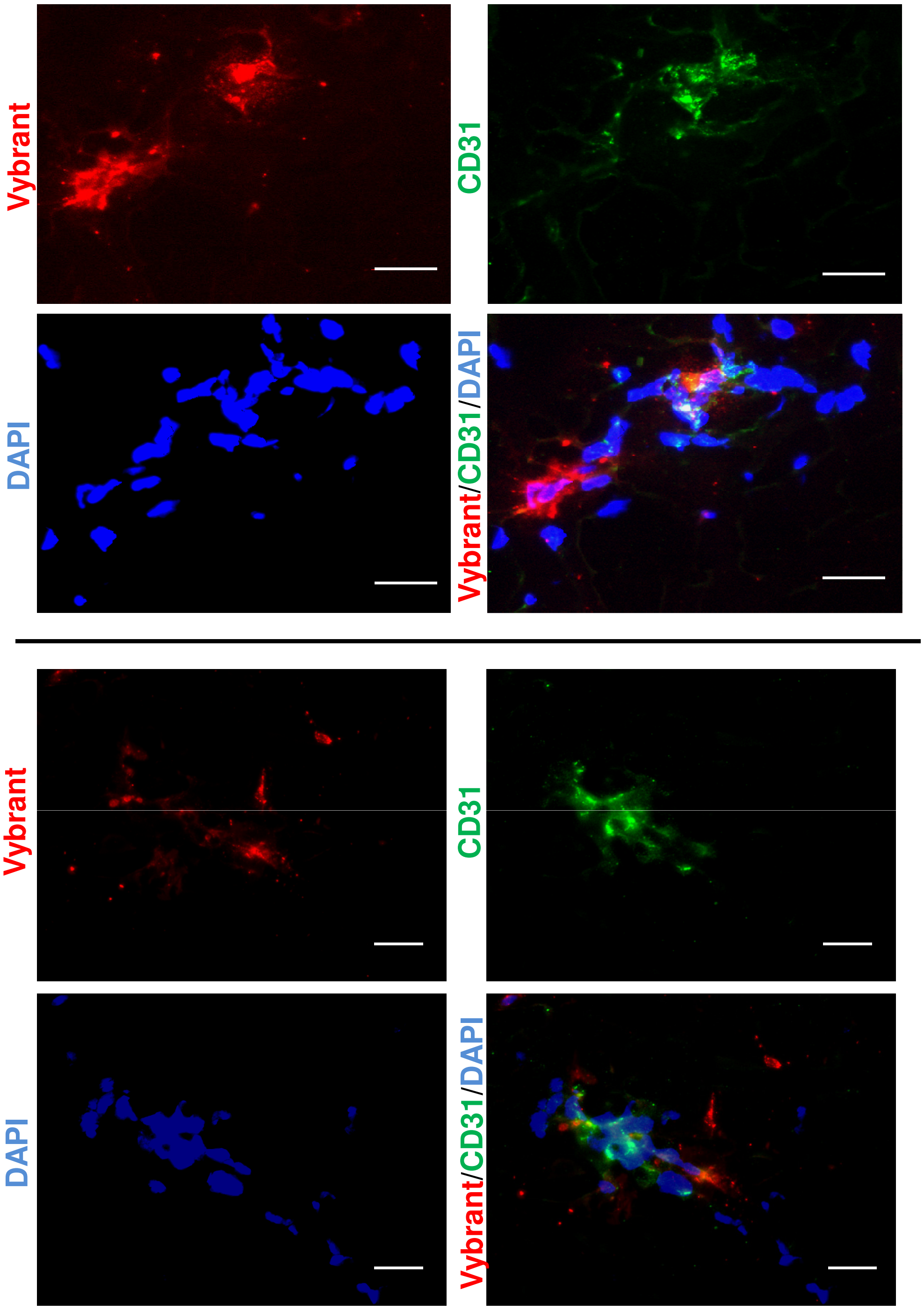

Supplementary Figure 12. Vybrant labeled SMC-CD34-ECs for *in vivo* Matrigel angiogenesis assay. Immunofluorescnece staining images showed that Vybrant labeled SMC-CD34-ECs participated in forming vascular-like structures in the *in vivo* Matrigel plug assays. (Scale bar: 25μm)

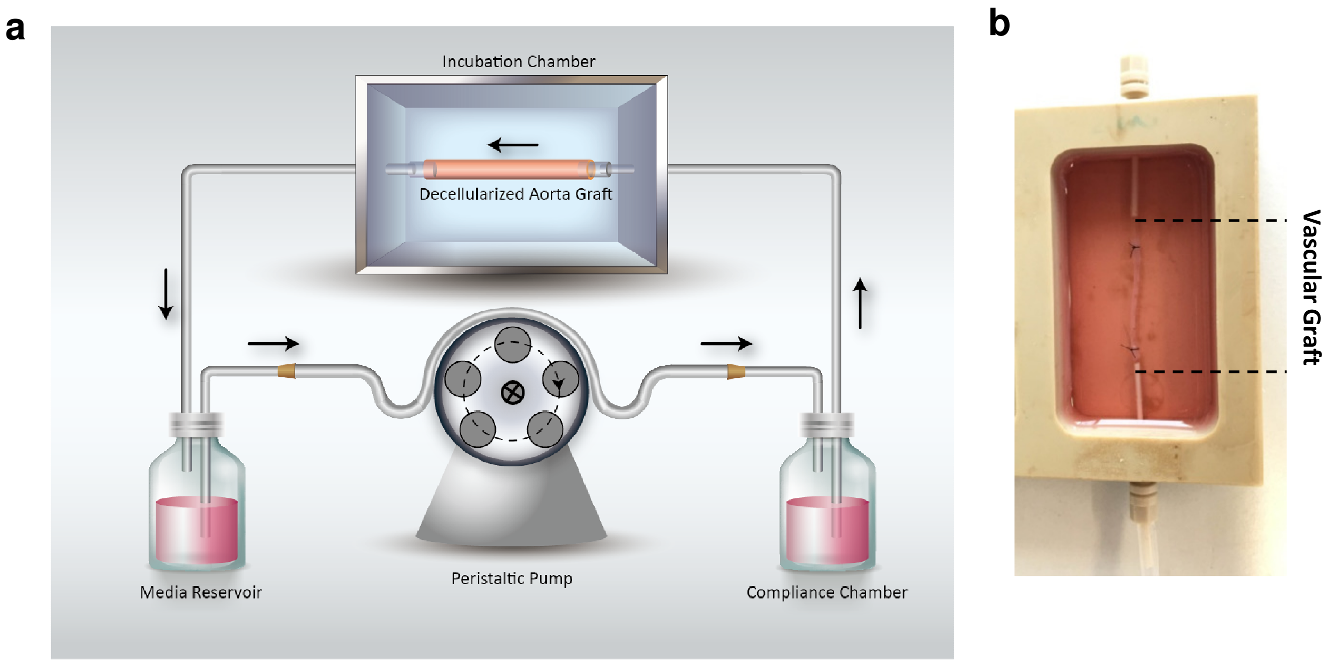

Supplementary Figure 13. Setting of the *ex vivo* bioreactor system to generate tissue-engineered vascular graft. (a) Schematic representation of the decellularized graft bioreactor flow circuit. The decellularized vascular graft is assembled in the incubation chamber. A peristaltic pump is at the upstream of the incubation chamber to provide stable medium perfusion flow. The media reservoir is at the downstream of the incubation chamber. The compliance chamber is to improve the flow regime. The flow direction is indicated by arrows. **(b)** Picture of a decellularized vascular graft assembled in the incubation chamber.

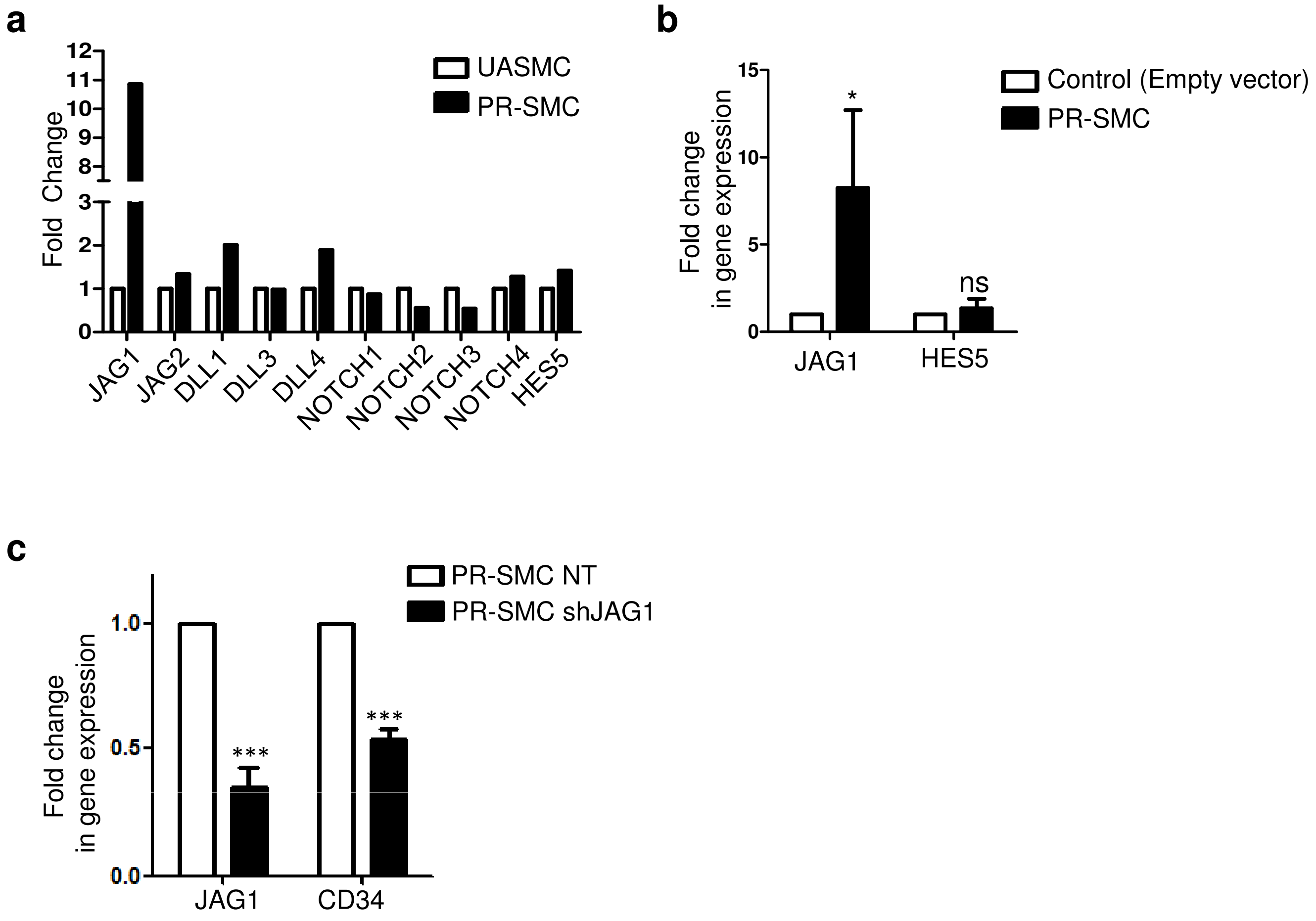

Supplementary Figure 14. JAG1 is involved in SMC to vascular progenitor state PR-SMCs conversion. (a) RNA-Seq data showed the gene expression fold change of Notch signaling pathway members in PR-SMCs compared to SMCs. **(b)** Real-time PCR analysis confirmed the upregulation of JAG1 in PR-SMCs compared to the control cells. SMCs transfected with empty lentiviral vector and kept under identical reprogramming conditions were used as the control group. (ns=non significant, *p<0.05 by Student's *t* test, n=3) **(c)** Real-time PCR analysis revealed that the knockdown of JAG1 impaired the upregulation of CD34 in PR-SMCs. (***p<0.001 by Student's *t* test, n=3).

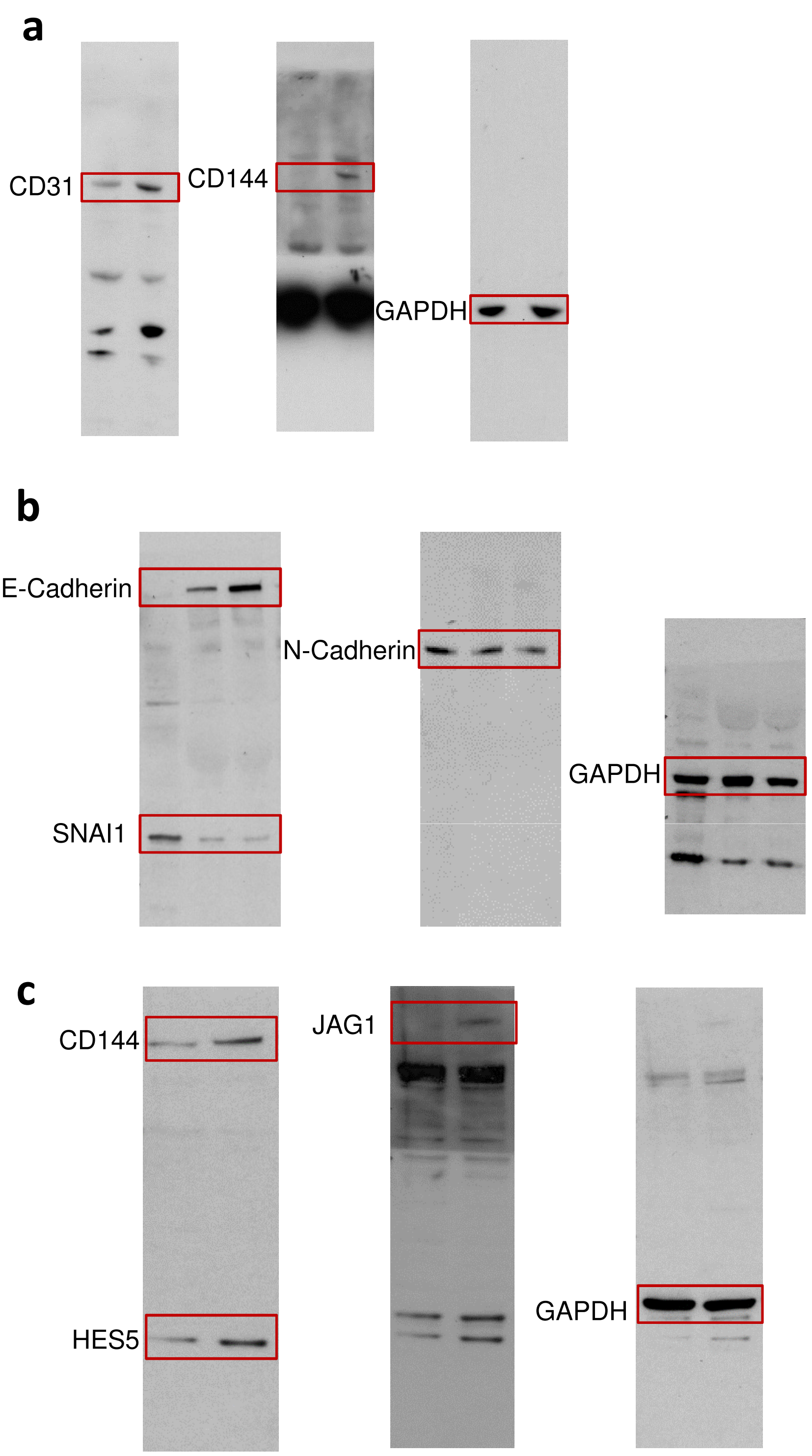

Supplementary Figure 15. Examples of Uncropped Western Blots . (a) Uncropped Western Blots for Figure 1g. **(b)** Uncropped Western Blots for Figure 5c. **(c)** Uncropped Western Blots for Figure 6c.

Table Supplementary I. Primers for real-time PCR

Name	Sequence (5'→3')
OCT4 Forward<br>OCT4 Reverse	ATGCATTCAAACTGAGGTGCCTGC<br>AACTTCACCTTCCCTCCAACCAGT
SOX2 Forward<br>SOX2 Reverse	CACATGAAGGAGCACCCGGATTAT<br>GTTCATGTGCGCGTAACTGTCCAT
KLF4 Forward<br>KLF4 Reverse	TGACCAGGCACTACCGTAAACACA<br>TCTTCATGTGTAAGGCGAGGTGGT
c-MYC Forward<br>c-MYC Reverse	ACAGCTACGGAACTCTTGTGCGTA<br>GCCCAAAGTCCAATTTGAGGCAGT
α-SMA Forward<br>α-SMA Reverse	TGACAATGGCTCTGGGCTCTGTAA<br>TTCGTCACCCACGTAGCTGTCTTT
SM22 α Forward<br>SM22 α Reverse	TTGAAGGCAAAGACATGGCAGCAG<br>TCCACGGTAGTGCCCATCATTCTT
Calponin Forward<br>Calponin Reverse	TTGAGGCCAACGACCTGTTTGAGA<br>TCGAATTTCCGCTCCTGCTTCTCT
SMMHC Forward<br>SMMHC Reverse	AGAAGCCAGGGAGAAGGAAACCAA<br>TGGAGCTGACCAGGTCTTCCATTT
CD34 Forward<br>CD34 Reverse	CACTGAGCAAGATGTTGCAAGCCA<br>TCAGGAAATAGCCAGTGATGCCCA
KDR Forward<br>KDR Reverse	ATCCAGTGGGCTGATGACCAAGAA<br>ACCAGAGATTCCATGCCACTTCCA
CD31 Forward<br>CD31 Reverse	AGCCCGAACTGGAATCTTCCTTCA<br>TCCTTCTGGATGGTGAAGTTGGCT
CD144 Forward<br>CD144 Reverse	GCCAGGTATGAGATCGTGGT<br>CAACAAACAGAGAGCCCACA
eNOS Forward<br>eNOS Reverse	ACCCTCACCGCTACAACATC<br>GCTCATTCTCCAGGTGCTTC

vwF Forward<br>vwF Reverse	CTGAAGGGCTCGAGTGTACC<br>CACATGGTCTGTGCAGTTCC
Claudin5 Forward<br>Claudin5 Reverse	CTGCTGGTTCGCCAACATT<br>TGCGACACGGGCACAG
E-Cadherin Forward<br>E-Cadherin Reverse	GTCACTGACACCAACGATAATCCT<br>CAGTGTGGTGATTACGACGTTA
SNAI1 Forward<br>SNAI1 Reverse	TCGGAAGCCTAACTACAGCGA<br>AGATGAGCATTGGCAGCGAG
Fibronectin Forward<br>Fibronectin Reverse	AAACTTGCATCTGGAGGCAAACCC<br>AGCTCTGATCAGCATGGACCACTT
Mucin1 Forward<br>Mucin1 Reverse	AGCTGCCCGTAGTTCTTTCG<br>CGCTGGCCATTGTCTATCTCA
Claudin1 Forward<br>Claudin1 Reverse	GCGCGATATTTCTTCTTGCAGG<br>TTCGTACCTGGCATTGACTGG
Jagged1 Forward<br>Jagged1 Reverse	GTCCATGCAGAACGTGAACG<br>GCGGGACTGATACTCCTTGA
HES5 Forward<br>HES5 Reverse	GTCAGCTACCTGAAGCACAGCAAA<br>TGGAAGTGGTACAGCAGCTTCATC
LIN28 Forward<br>LIN28 Reverse	AGCGCAGATCAAAAGGAGACA<br>CCTCTCGAAAGTAGGTTGGCT
REX1 Forward<br>REX1 Reverse	TGAAAGCCCACATCCTAACG<br>CAAGCTATCCTCCTGCTTTGG
NANOG Forward<br>NANOG Reverse	CCCAAAGGCAAACAACCCACTTCT<br>AGCTGGGTGGAAGAGAACACAGTT
GAPDH Forward<br>GAPDH Reverse	CATGTTCGTCATGGGTGTGAACCA<br>ATGGCATGGACTGTGGTCATGAGT
